# A Mathematical Model for Electrical Activity in Pig Atrial Tissue

**DOI:** 10.3389/fphys.2022.812535

**Published:** 2022-03-10

**Authors:** Víctor Peris-Yagüe, Tony Rubio, Funsho E. Fakuade, Niels Voigt, Stefan Luther, Rupamanjari Majumder

**Affiliations:** ^1^Biomedical Physics Group, Max Planck Institute for Dynamics and Self Organisation, Gottingen, Germany; ^2^Centre de Formaćio Interdisciplinària Superior (CFIS), Universitat Politècnica de Catalunya, Barcelona, Spain; ^3^Institute of Pharmacology and Toxicology, University Medical Center Göttingen, Georg-August University, Gottingen, Germany; ^4^German Center for Cardiovascular Research (DZHK), Partner Site Göttingen, Gottingen, Germany; ^5^Cluster of Excellence “Multiscale Bioimaging: From Molecular Machines to Networks of Excitable Cells” (MBExC), Georg-August University, Gottingen, Germany

**Keywords:** ionic model, pig atria, spiral waves, large animal, cardiac electrophysiology

## Abstract

State of the art mathematical models are currently used to bridge the gap between basic research conducted in the laboratory and preclinical research conducted on large animals, which ultimately paves the way for clinical translation. In this regard, there is a great need for models that can be used alongside experiments for in-depth investigation and validation. One such experimental model is the porcine atrium, which is commonly used to study the mechanisms of onset and control of atrial fibrillation in the context of its surgical management. However, a mathematical model of pig atria is lacking. In this paper, we present the first ionically detailed mathematical model of porcine atrial electrophysiology, at body temperature. The model includes 12 ionic currents, 4 of which were designed based on experimental patch-clamp data directly obtained from literature. The formulations for the other currents are adopted from the human atrial model, and modified for porcine specificity based on our measured restitution data for different action potential characteristics: resting membrane potential, action potential amplitude, maximum upstroke velocity and action potential duration and different levels of membrane voltage repolarization. The intracellular *Ca*^2+^ dynamics follows the Luo-Rudy formulation for guinea pig ventricular cardiomyocytes. The resulting model represents “normal” cells which are formulated as a system of ordinary differential equations. We extend our model to two dimensions to obtain plane wave propagation in tissue with a velocity of 0.58 m/s and a wavelength of 8 cm. The wavelength reduces to 5 cm when the tissue is paced at 200 ms. Using S1-S2 cross-field protocol, we demonstrate in an 11.26 cm square simulation domain, the ability to initiate single spiral waves (rotation period ≃ 180 ms) that remain stable for more than 40 s. The spiral tip exhibits hypermeander. In agreement with previous experimental results using pig atria, our model shows that early repolarization is primarily driven by a calcium-mediated chloride current, *I*_*ClCa*_, which is completely inactivated at high pacing frequencies. This is a condition that occurs only in porcine atria. Furthermore, the model shows spatiotemporal chaos with reduced repolarization.

## 1. Introduction

Atrial fibrillation (AF) is the most common sustained form of cardiac arrhythmia occurring in humans. Its effective treatment requires a detailed understanding of the underlying mechanisms at the genetic, molecular, cellular, tissue and organ levels. To study the complex mechanisms underlying the development, maintenance and termination of cardiac arrhythmias, preclinical research models are required. These models range from *in vitro* cell cultures to *in vivo* small and large animal hearts. However, translational research necessitates a proper understanding of the results from animal experiments in the human context, for which it is very important that the preclinical results are well-understood and validated. Currently, this is achieved through simulations of state-of-the-art mathematical models alongside experimentation on large animals. In particular, a model that is extensively used by experimentalists to advance surgical management of AF, is that of the pig atria. However, until now, an ionically detailed mathematical model for pig atrial tissue has been lacking, and researchers have been forced to rely on mathematical models from other animal species to understand their experimental observations.

Typical large animal heart models include that of dog, sheep, goat, pig, etc. In studies on cardiac arrhythmias, especially atrial fibrillation (AF) it is a challenge to find the right animal model. This is mainly because the reliable inducibility of sustained AF requires some form of chronic intervention, which is often associated with a large cost of maintenance and time limitations. The porcine atrial model overcomes this challenge by providing an acute, reliable and reproducible model for sustained AF (Lee et al., 2016). This model can be used at length to test experimental procedures and drugs that are intended for translational purposes under various disease conditions. To make matters more favorable, the cardiac anatomy, electrophysiology and coronary circulation of pigs are very similar to those of humans (e.g., heart mass: 148–383 g in humans vs. 250–400 g in pigs; heart to body mass ratio: 0.4 in humans vs. 0.32 in pigs; heart rate: 60–80 in humans vs. 68–100 in pigs, etc.) For a detailed comparison of human and pig parameters, we refer the reader to Table 1 of Clauss et al. (2020). Thus, an electrophysiologically detailed mathematical model of the porcine atria is definitely a important tool to have in translational research.

In this study, we present the first detailed mathematical model of the pig atria, based on experimental patch-clamp data from literature and our own restitution experiments on pig atrial tissue, using sharp-electrode technique. In two dimensions, we demonstrate its ability to produce and sustain stable meandering spiral waves. We characterize the spatiotemporal meander pattern, report the dominant frequencies and the effect of system size on the stability of the spiral pattern. In particular, we highlight some fundamental differences in the role of Cl^−^ currents and Ca^2+^ dynamics in the early repolarisation phase of AP in pigs with respect to humans, a crucial difference to be accounted for in the translatability of results, from pigs to humans, in future *in vitro* and *in vivo* experiments. We further go on to propose a model for AF using an altered set of parameters that allows us to have a state of electric turbulence in pig atrial tissue.

## 2. Materials and Methods

All animal care and use procedures were carried out exclusively by appropriately trained staff and were in accordance with the German Animal Welfare Act and reported to the local animal welfare officers. The handling of the animals prior to the experiments and the humane, animal welfare procedures strictly followed animal welfare regulations, in accordance with German legislation, local regulations and the recommendations of the Federation of European Laboratory Animal Science Associations (FELASA). All scientists and technicians involved have been accredited by the responsible ethics committee (Lower Saxony State Office for Consumer Protection and Food Safety - LAVES).

###  Experimental Recordings

Trabecular muscles were isolated and excised from a whole right atria, and placed in a custom-built recording chamber under continuous perfusion of heated (37°C) and carbonated (5% CO_2_, 95% O_2_) Tyrode's solution containing (in mM): NaCl 126.7, KCl 5.4, MgCl_2_ 1.1, CaCl_2_ 1.8, NaHPO_4_ 0.42, NaHCO_3_ 22, glucose 5.5, pH = 7.45 at least 45 min before measurement, for accommodation.

Borosilicate glass capillaries (Hilgenberg, Germany) were pulled using a horizontal pipette puller (Zeitz, Germany). Electrical resistance was 30–40 MΩ. Pipettes were backfilled with 3M KCl.

Tissues were electrically stimulated with a 1 ms monophasic pulse using a custom-made electrode (FHC, USA). Pulse amplitude was pre-defined as 30% higher than the value necessary to trigger an action potential. After successful tissue impalement, and after reaching steady state activity, the tissue was then subjected to a train of electrical stimulation at increasing frequencies (0.25, 0.5, 1, 2, 3, and 4 Hz). AP onset at 5 Hz proved difficult and inconsistent.

Membrane potential signals were amplified using a Sec-05-X (npi, Germany) amplifier, digitized using LabChart PowerLab, and acquired and saved with LabChart Pro 7 software (both: ADInstruments, New Zealand).

Analysis was performed using LabChart pro and GraphPad Prism 7 (GraphPad Software Inc., USA). The average value of 10 consecutive action potentials were calculated in LabChart Pro. The following parameters were measured: resting membrane potential (RMP), action potential maximum upstroke velocity (dV/dtmax), action potential amplitude (APA) and the action potential duration at 20, 50 and 90% of repolarisation (*APD*_20_, *APD*_50_, and *APD*_90_, respectively).

###  Mathematical Model

The fitting of IV curves from experimental data by Li et al. (2004) was carried out by minimization of the squared error between the simulated and experimental data using Python's Scipy module (Virtanen et al., 2020). For this purpose, a function was created in Python that would recreate the patch-clamp experiments as in Li et al. (2004), and output the simulated IV curve. The morphology of each individual current and its gating variables was initially taken directly from the human atrial model by Courtemanche et al. (1998) and corrected accordingly to match experimental data. Fitting was done by matching normalized IV curves first, and then adjusting conductance values to match the non-normalized experimental IV curves.

Overall AP morphology and restitution curves were later matched by re-adjusting conductance values of the different currents, and simulating AP evolution for stimulation at different cycle lengths. Slight changes were made to currents not fitted from Li et al. (2004) to better adjust experimental restitution curves. See the Results section for detailed explanations of each individual current.

## 3. Results

**Figure 1:**
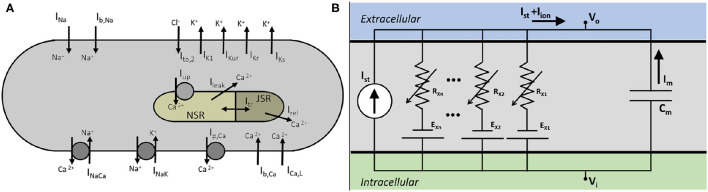
**(A)** Schematics of a pig atrial cardiomyocyte model, showing transmembrane currents and the basic structure of the *Ca*^2+^ dynamics. **(B)** Electrical circuit equivalent of the cell.

We developed a mathematical model for a native atrial cardiomyocyte, isolated from the excised atrium of a healthy adult pig. The equivalent electrical circuit representing the cell membrane is shown in Figure 1.

It consists of a membrane capacitance, *C*_*m*_, connected in parallel with several nonlinear conductances (*G*_*X*_) and batteries (*E*_*X*_). The net current (*I*_*ion*_) flowing across the cell membrane at any instant is the sum of individual currents flowing through the various branches of the circuit. Thus, the time-evolution of the transmembrane voltage *V* can be described using the following ordinary differential equation. (Equation 1)


(1) $$\begin{array}{r} \frac{dV}{dt}=-\frac{I_{ion}+I_{stim}}{C_{m}} \end{array}$$

Here *I*_*stim*_ represents the external stimulus current that needs to be applied to the cell membrane to invoke an action potential (AP). We describe *I*_*ion*_ as a sum of 12 ionic currents (Equation 2):


(2) $$\begin{array}{c} I_{ion}=I_{Na}+I_{K1}+I_{ClCa}+I_{Kur}+I_{Kr}+I_{Ks}\\ \;+I_{Ca,L}+I_{p,Ca}+I_{NaK}\\ \;+I_{NaCa}+I_{b,Na}+I_{b,Ca} \end{array}$$

*V* is measured in millivolts (mV), time (*t*) in milliseconds (ms), *C*_*m*_ in picofarads (pF), and all currents in picoamperes per picofarad (pA/pF). All conductances *G*_*X*_ are measured in nanosiemens per picofarad (nS/pF), and intracellular and extracellular ionic concentrations ([*X*]_*i*_, [*X*]_*o*_) are expressed in milimolar (mM). The fast *Na*^+^ current is represented by *I*_*Na*_, the inward rectifier *K*^+^ current by *I*_*K*1_, ultrarapid rectifier *K*^+^ current is given by *I*_*Kur*_, rapid and slow delayed rectifier *K*^+^ currents by *I*_*Kr*_, *I*_*Ks*_, respectively, *L-Type* *Ca*^2+^ current by *I*_*Ca, L*_, *Ca*^2+^ pump current by *I*_*p, Ca*_, the Sodium-Potassium and Sodium-Calcium pump currents by *I*_*NaK*_, *I*_*NaCa*_, respectively, and the background *Na*^+^ and *Ca*^2+^ currents by *I*_*b, Na*_, *I*_*b, Ca*_, respectively. Uniquely in the pig atrial model, the transient outward current is represented only by a calcium-mediated chloride current, *I*_*ClCa*_.

The formulation of subcellular *Ca*^2+^ uptake and release by the sarcoplasmic reticulum (SR) is retained from the work of Luo and Rudy (1994). The three main currents involved are the *Ca*^2+^ uptake current, *I*_*up*_, the *Ca*^2+^ release current *I*_*rel*_ and the *Ca*^2+^ transfer current between the network SR (NSR) and junctional SR (JSR), *I*_*tr*_. The model also includes a leak current from the SR into the cytoplasm, *I*_*up, leak*_, as described by Courtemanche et al. in their model for the human atrial cardiomyocyte (Courtemanche et al., 1998).

To invoke action potentials in tissue, we applied a stimulus current of 7 nA for 4 ms. The mathematical description of each ionic current is provided in the Supplementary Appendix, together with a list of model parameters and initial values.

###  Membrane Currents

#### Fast Sodium, *I*_*Na*_

We describe the fast *Na*^+^ current according to the Courtemanche-Ramirez-Nattel (CRN) model for human atrial cardiomyocytes (Courtemanche et al., 1998), which uses a Hodgkin-Huxley type formulation (see Equation 3) taken from the Luo-Rudy model (Luo and Rudy, 1994):


(3) $$\begin{array}{r} I_{Na}=g_{Na}m^{3}hj\left(V-E_{Na}\right) \end{array}$$

Here *m* is the activation gate; *h* and *j* are the two inactivation gates. In order to make the model pig-specific, we used Equation (3) to fit experimentally obtained *I*_*Na*_ current-voltage (IV) characteristics and/or current traces from patch-clamp measurements.

However, in the absence of these experimental data, we followed an alternative approach. Since *I*_*Na*_ is the dominant active current during the upstroke phase of an AP, (the other current being the inward rectifier, *I*_*K*1_, which is orders of magnitude smaller than *I*_*Na*_), we considered it to be primarily responsible for the AP amplitude (APA) and maximum upstroke velocity (${\frac{dV}{dt}}_{max}$). Thus, we used the complete model (considering all currents, pumps and exchangers) to fit experimentally obtained APA and ${\frac{dV}{dt}}_{max}$ data, at various pacing frequencies, i.e., APA- and ${\frac{dV}{dt}}_{max}$ restitution, by tuning only the *I*_*Na*_.

**Figure 2:**
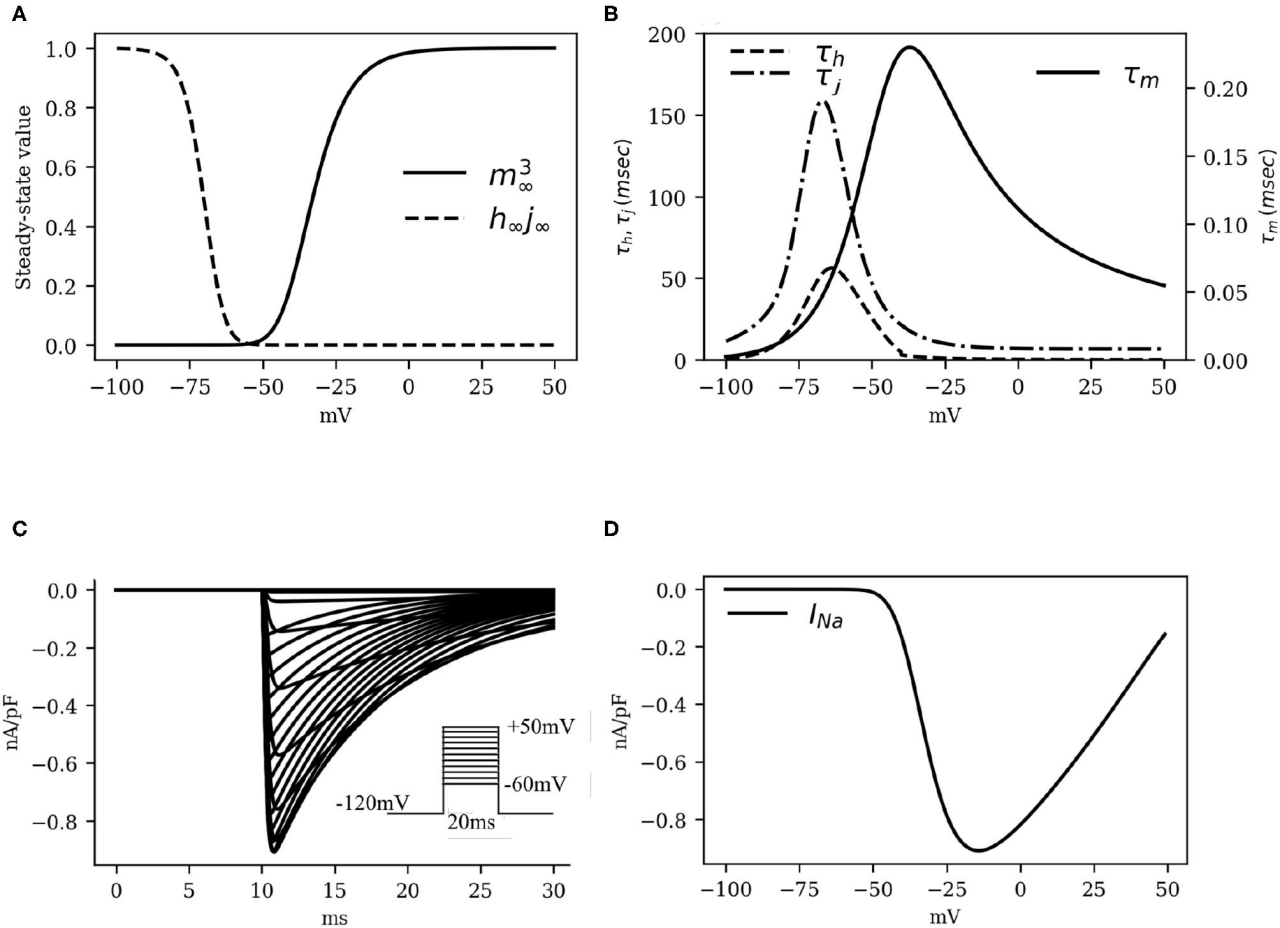
The kinetics of the fast *Na*^+^ current *I*_*Na*_. **(A)** Activation and inactivation characteristics of the steady state gating variables *m* (raised to cubic power), *h* and *j* (combined as the product *h*_∞_*j*_∞_). **(B)** Voltage dependence of the time constants for activation (τ_*m*_) and fast and slow inactivation (τ_*h*_ and τ_*j*_, respectively). **(C)** Simulated traces of *I*_*Na*_ (traces at 5*mV* steps from minimum to maximum in the inset). **(D)** *I*_*Na*_ Current-Voltage characteristics.

Numerical fitting of these restitution data, using Equation (3) to describe *I*_*Na*_ instructed us to apply the following adaptations: (*i*) raise the maximum channel conductance (*g*_*Na*_) by 80% with respect to humans; (*ii*) increase the time constant of activation (τ_*m*_) by a factor 1.7; and (*iii*) increase the time constant of inactivation (τ_*h*_, τ_*j*_) by a factor 2. The kinetics of the activation and inactivation gates are shown in Figures 2A,B. With the applied modifications, the *I*_*Na*_ current traces turned out to be as in Figure 2C and the IV curve, as shown in Figure 2D.

#### L-Type Calcium Current, *I*_*Ca, L*_

The L-type *Ca*^2+^ currrent (*I*_*Ca, L*_) was modeled according to previous literature (Courtemanche et al., 1998; Ramirez et al., 2000; Pandit et al., 2001; Majumder et al., 2016), based on the Hodgkin-Huxley formalism:


(4) $$\begin{array}{r} I_{Ca,L}=g_{Ca,L}dff_{Ca}\left(V-65.0\right) \end{array}$$

Here *g*_*Ca, L*_ is the channel conductance, *d* and *f* the voltage-gated activation and inactivation variables, respectively, and *f*_*Ca*_ is a calcium-mediated gating variable defined by:


(5) $$\begin{array}{r} \tau_{f\left(Ca\right)}=2,\;f_{Ca,\infty }=\frac{1}{1+\frac{{\left[Ca^{2+}\right]}_{i}}{0.00035}} \end{array}$$

**Figure 3:**
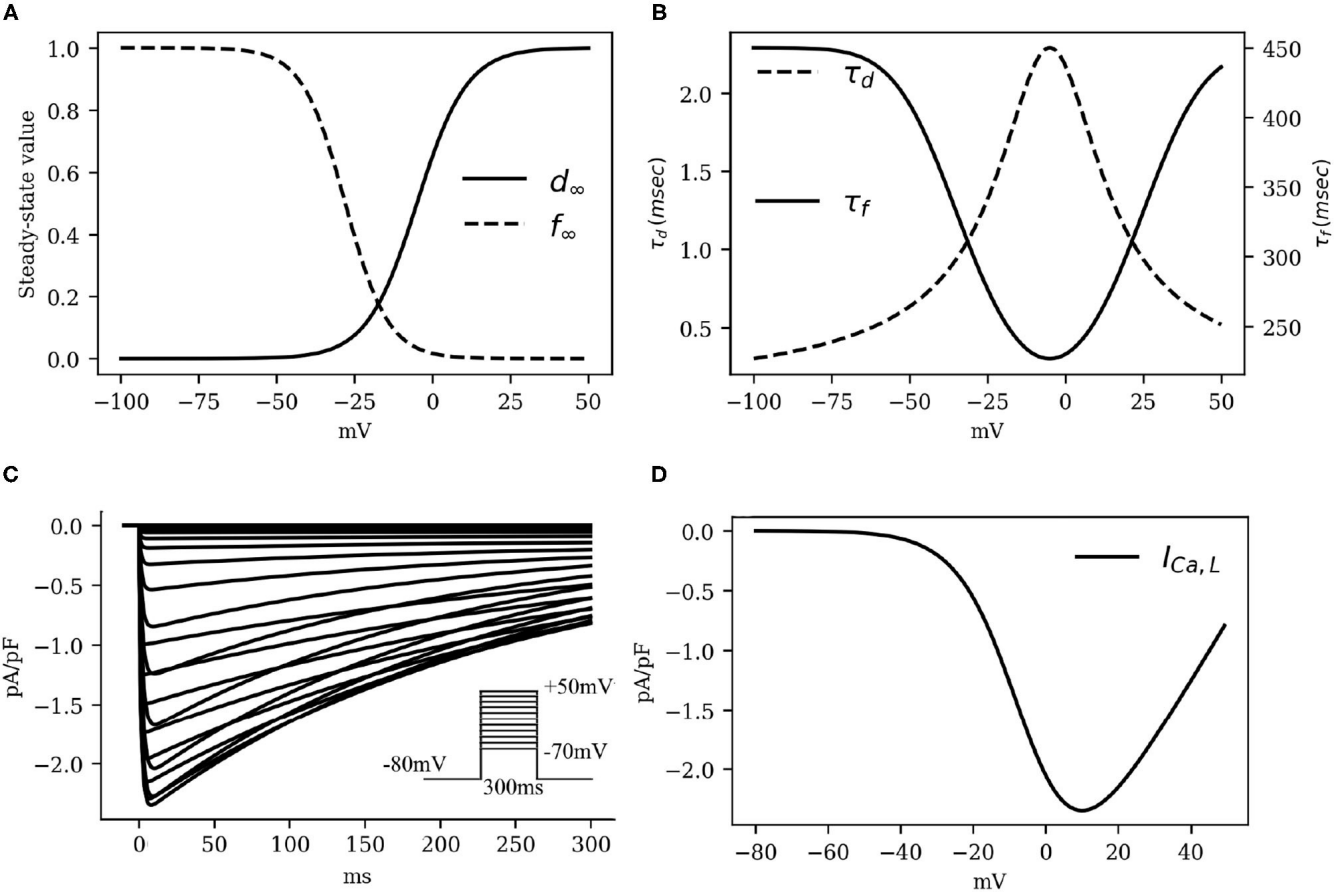
The kinetics of the fast L-type *Ca*^2+^ current *I*_*CaL*_. **(A)** Activation and inactivation characteristics of the steady state gating variables *d* and *f*. **(B)** Voltage dependence of the time constants for activation (τ_*d*_) and voltage-gated inactivation (τ_*f*_). **(C)** Simulated traces of *I*_*CaL*_ (traces at 5*mV* steps from minimum to maximum in the inset). Intracellular *Ca*^2+^ concentration was kept constant at ${\left[Ca^{2+}\right]}_{i}=0.0001mM$. **(D)** *I*_*CaL*_ Current-Voltage characteristics.

In particular, the gating behavior of *I*_*Ca, L*_ follows the human CRN model, with a +5*mV* shift in the activation kinetics to decrease the activation window along with the overall *I*_*Ca, L*_ that is necessary for the fitting of action potential duration restitution properties. As *I*_*Ca, L*_ is considered to be largely responsible for the plateau phase of the action potential, decreasing (increasing) this current by small amounts can lead to sharp decrease (increase) in the action potential duration without lowering (raising) the resting membrane potential by substantial amounts. Our patch-clamp measurements showed that the amplitude of *I*_*Ca, L*_ was ≃3.25±0.75 pA/pF. This value imposed a constraint on the choice of *g*_*CaL*_. The kinetics of L-type *Ca*^2+^ channel, as well as the IV characteristics of the *I*_*CaL*_ are shown in Figure 3.

#### Inward Rectifier Potassium Current, *I*_*K*1_

The *I*_*K*1_ is known to play a major role in determining the resting membrane potential (RMP) of excitable cardiac cells in many animal species, with the current reversing its direction of flow close to the actual RMP value. Given that our sharp-electrode measurements on pig atrial tissue suggested a more depolarised (positive) RMP value than that reported in human atrial cardiomyocytes (Courtemanche et al., 1998), we modified the parameters of the CRN *I*_*K*1_ formulation to make the current pig-specific.

**Figure 4:**
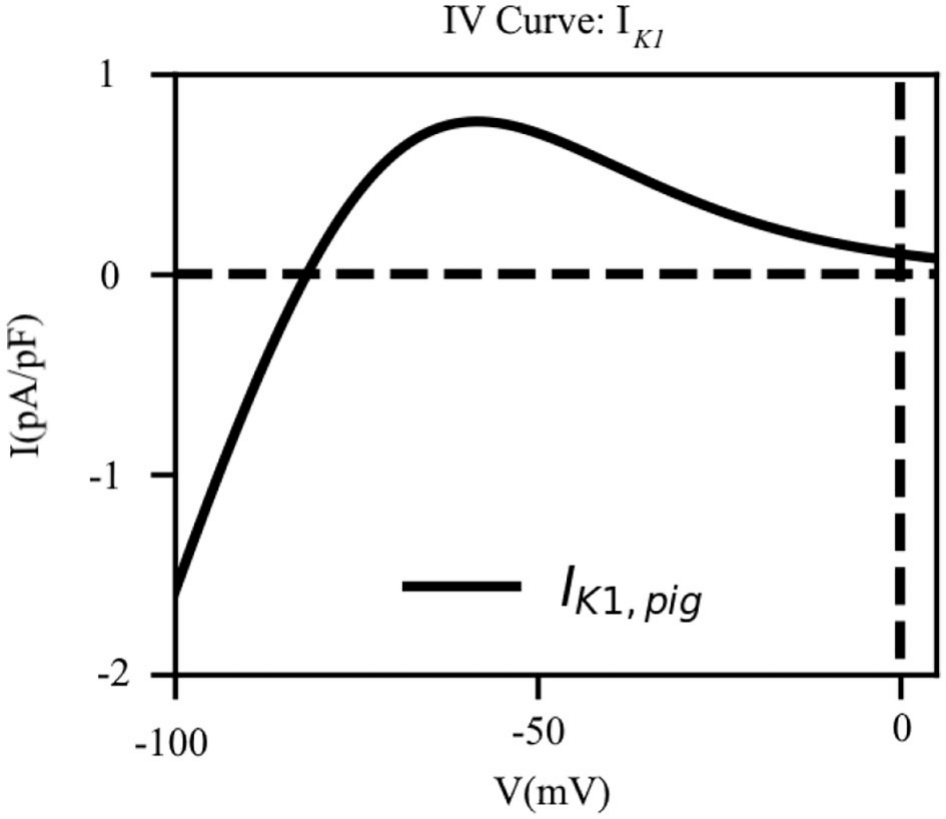
The current-voltage characteristic of the inward rectifier *K*^+^ current (*I*_*K*1_). The direction of flow of the current across the cell membrane reverses at *E*_*K*_ = −81.76*mV* which is close to our experimentally measured resting membrane potential.

To this end, (*i*) we shifted the reversal potential of *I*_*K*1_ by –5 mV, as has been done previously in some large animal models, such as the sheep (Butters et al., 2013), (*ii*) we reduced the maximum channel conductance of *I*_*K*1_ by 9% relative to the human model (Courtemanche et al., 1998), (*iii*) we decreased the slope of activation of the *I*_*K*1_ IV curve by 10%; and (*iv*) we shifted the half-rise potential by +10 *mV*. Thus, *I*_*K*1_ in the pig atrial model is described according to Equation (6). These adjustments enabled us to fit the shape of the action potential duration restitution curves at the 80–90% repolarization, while trying to balance the effects of *I*_*Ca, L*_ and other rectifier currents. The IV curve for *I*_*K*1_ as shown in Figure 4.


(6) $$\begin{array}{r} I_{K1}=g_{K1}\cdot \frac{V-E_{K}-5}{1+exp\left(0.063\cdot \left(V+70\right)\right)} \end{array}$$

#### Ultrarapid Potassium Current, *I*_*Kur*_

Recent work by Ehrlich et al. (2006), indicates that pig atrial tissue exhibits a bi-exponential inactivation. Pandit et al. (2011) developed a model for the ultrarapid *K*^+^ current, that reproduces the experimental data of Ehrlich et al. (2006). We used the formulation of Pandit et al. (2011) to describe *I*_*Kur*_ in our model for the pig atrial tissue (see Equation 7)


(7) $$\begin{array}{r} I_{Kur}=g_{Kur}\cdot u_{a}^{3}\cdot \left(au_{i,f}+bu_{i,s}\right)\cdot \left(V-E_{K}\right) \end{array}$$

Here *g*_*Kur*_ is the channel conductance, *u*_*a*_ the activation gate, *E*_*K*_ the reversal potential of *K*^+^, and *u*_*i, f*_ and *u*_*i, s*_ the fast and slow inactivation components, respectively. (*a, b*) = (0.25, 0.75) are weights applied to the inactivation gates. It is interesting to note that the approach by Pandit et al. is similar to that of Aguilar et al. for the human atria. However, in the latter case, the inactivation of *I*_*Kur*_ is given by *u*_*i*_ = *u*_*i, f*_·*u*_*i, s*_ instead of a sum of the variables (Aguilar et al., 2017). The conductance of *I*_*Kur*_ is described according to Equation (8).


(8) $$g_{Kur}=g_{Kur,amp}\left[0.005+\frac{0.05}{1+\exp(-\frac{V-15}{13})}\right]$$

**Figure 5:**
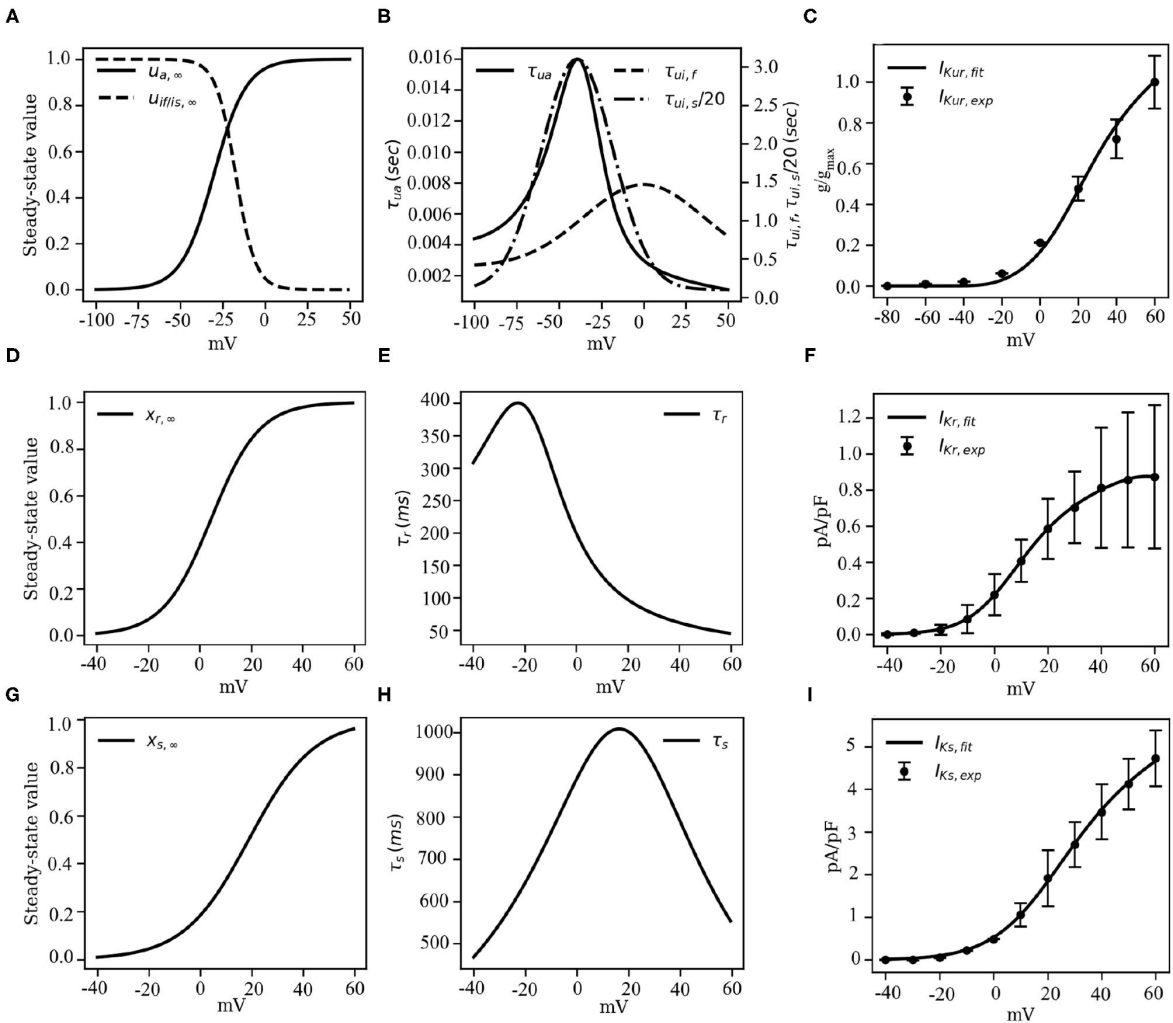
Channel kinetics and current-voltage characteristics of the rectifying *K*^+^ currents *I*_*Kur*_, *I*_*Kr*_ and *I*_*Ks*_. **(A)** Voltage-dependence of steady-state activation (*u*_*s*, ∞_) and inactivation (*u*_*if*, ∞_ of *u*_*is*, ∞_) probabilities of *I*_*Kur*_. **(B)** Voltage-dependence of the time constants of activation (τ_*ui*_) and inactivation (τ_*uf*_ or τ_*us*_) of *I*_*Kur*_, reduced by a factor of 20. **(C)** Comparison between the model-generated IV curve for *I*_*Kur*_ and the IV curve reported by Ehrlich et al. (2006) based on experiments. **(D)** Voltage-dependence of steady-state activation (*x*_*r*, ∞_) probability of *I*_*Kr*_. **(E)** Voltage-dependence of the time constant of activation (τ_*r*_) of *I*_*Kr*_. **(F)** Comparison between the model-generated IV curve for *I*_*Kr*_ and the experimentally obtained IV curve from Li et al. (2004). **(G)** Voltage-dependence of steady-state activation (*x*_*s*, ∞_) probability of *I*_*Ks*_. **(H)** Voltage-dependence of the time constant of activation (τ_*s*_) of *I*_*Ks*_. **(I)** Comparison between the model-generated IV curve for *I*_*Ks*_ and the experimentally obtained IV curve from Li et al. (2004).

Here *g*_*Kur, amp*_ is an adjustable parameter whose value is determined during the final stages of model development (see section 3.2, for more details). Figures 5A,B show the activation and inactivation kinetics of *I*_*Kur*_, whereas, a comparison between the current-voltage characteristics, as measured in experiments by Ehrlich et al. (2006) and that produced using our model, is presented in Figure 5C.

#### Rapid Delayed Rectifier Current, *I*_*Kr*_

The rapid delayed rectifier current (*I*_*Kr*_) was formulated similar to the original CRN model (Courtemanche et al., 1998), but with altered half-rise voltage (*V*_1/2_), slope of the correction value, and the steady-state of the single gating variable, such that the obtained IV characteristic curve matches with the experimental IV curve of Li et al. (2004):


(9) $$\begin{array}{r} I_{Kr}=g_{Kr}\cdot x_{r}\cdot \frac{V-E_{K}}{1+exp\left(\frac{V-79.4825}{8.2217}\right)} \end{array}$$

Initially, the conductance *g*_*Kr*_ was set at 0.0065*pA*/*pF* to match the non-normalized IV curve. However, this value was tuned during the final stages of model development to match the pig atrial APD restitution curves at the tissue level. The steady-state value (*x*_*r*, ∞_) of the gating variable *x*_*r*_ is described according to Equation (10):


(10) $$\begin{array}{r} x_{r,\infty }=\frac{1}{1+exp\left(\frac{-\left(V-4.4451\right)}{9.3305}\right)} \end{array}$$

Note that, *V*_1/2_ of *x*_*r*, ∞_ is shifted by +20*mV* relative to the CRN model, whereas, the slope of the *x*_*r*_ kinetic is slightly decreased (Courtemanche et al., 1998). The gating behavior of *x*_*r*_ is described in Figures 5D,E. Unavailability of sufficient experimental data led us to retain the temporal dynamics of the gating variable *x*_*r*_ from the CRN model (Courtemanche et al., 1998). Figure 5F shows the comparison between the experimental and simulated IV curves for *I*_*Kr*_.

#### Slow Delayed Rectifier Current, *I*_*Ks*_

We retained the slow delayed rectifier current formulation from the original CRN model (Courtemanche et al., 1998):


(11) $$\begin{array}{r} I_{Ks}=g_{Ks}\cdot x_{s}^{2}\cdot \left(V-E_{K}\right) \end{array}$$

The maximum channel conductance *g*_*Ks*_ was adjusted to fit the restitution properties. The gating variable *x*_*s*_, and time constant τ_*s*_ are described according to Equations (12) and (13), respectively.


(12) $$x_{s,\infty }={\left[\frac{1}{1+exp(-\frac{V-p_{1}}{p_{2}})}\right]}^{1/2}$$

(13) $$\tau_{s}=\frac{1}{2}\;\cdot \;\left[\frac{1}{4\times 10^{-5}\;\cdot \;\frac{\left(V-p_{1}\right)}{1-exp(-\frac{V-p_{1}}{17})}+3.5\times 10^{-5}\;\cdot \;\frac{\left(V-p_{1}\right)}{exp(\frac{V-p_{1}}{9})-1}}\right],$$

Here, parameters *p*_1_ and *p*_2_ have values 18.802 and 12.6475 mV, respectively, obtained by fitting experimental data from Li et al. (2004). The close resemblance of these values with those used in the human atrial tissue model (Courtemanche et al., 1998) suggests that electrophysiologically, human atrial *I*_*Ks*_ and pig atrial *I*_*Ks*_ are very similar. Figures 5G,H show the steady state kinetic and time constant, respectively, of *I*_*Ks*_, whereas, the model-generated IV curve is compared with experimental data from Li et al. in Figure 5I.

#### Transient Outward Current, *I*_*to*_

*I*_*to*_ in most species is composed of two components: a potassium current (*I*_*to*, 1_) and a chloride current (*I*_*to*, 2_, also referred to as *I*_*ClCa*_).


(14) $$\begin{array}{r} I_{to}=I_{to,1}+I_{to,2}=I_{to,1}+I_{ClCa} \end{array}$$

Although the presence of *I*_*ClCa*_ has been reported in multiple species and tissues (Zygmunt and Gibbons, 1991; Gomis-Tena and Saiz, 1998; Ramirez et al., 2000; Xu et al., 2002; Bondarenko et al., 2004; Wang and Sobie, 2008), including human atria (Wang et al., 1987, 1993), it is generally observed that *I*_*to*, 1_ forms the predominant component, scoring over *I*_*ClCa*_ in both strength and duration of activity, as a transient outward current (Wang et al., 1993; Bondarenko et al., 2004). However, in a study by Li et al. (2004), it was reported that pig atrial *I*_*to*_ is unique, in the sense that it is a completely calcium-driven chloride current (Li et al., 2003, 2004; Schultz et al., 2007). Thus, in our model, we incorporated this feature by modeling *I*_*ClCa*_ according to Equation (15), and experimental data from Li et al. (2004).


(15) $$\begin{array}{r} I_{to}\equiv I_{ClCa}=g_{ClCa}\cdot q_{Ca}\left(V-E_{Cl}\right) \end{array}$$

For the choice of formulation of *I*_*ClCa*_ we considered various candidates (Gomis-Tena and Saiz, 1998; Ramirez et al., 2000; Bondarenko et al., 2004; Wang and Sobie, 2008). In the end, we decided to use Equation (15), which is a formulation for *I*_*ClCa*_ in a canine atrial model (Ramirez et al., 2000). The reason for choosing this formulation was that it allowed a fairly accurate reproduction of the bell shape of the IV curve and the same general upward trend present in the experimental data of Li et al. (2004).

Here *g*_*ClCa*_ is the channel conductance, *E*_*Cl*_ the *Cl*^−^ reversal potential and *q*_*Ca*_ the sole gating variable of the channel, which follows the typical gating behavior of a Hodgkin-Huxley-type gating variable:


(16) $$\begin{array}{l} q_{Ca}(t)=q_{Ca,\infty }-(q_{Ca,\infty }-q_{Ca,0})\cdot e^{-\frac{t-t_{0}}{\tau_{Ca}}}\\ q_{Ca,\infty }=1-\left[\frac{1}{1+{\left(\frac{F_{n}}{1.1e-10}\right)}^{3}}\right],\;\tau_{Ca}=2 \end{array}$$

*F*_*n*_ is the flux of *Ca*^2+^ into the myoplasm. *F*_*n*_ shows a strong correlation with the sharp release of *Ca*^2+^ from the SR in the initial stages of AP (through the SR release current, *I*_*rel*_), giving *I*_*ClCa*_ the fast dynamics of a transient outward current. Also, the inactivation of *I*_*rel*_ gives *I*_*ClCa*_ a significant bell-shape in its IV curve, something universally observed in *I*_*ClCa*_ (Tseng and Hoffman, 1989; Gomis-Tena and Saiz, 1998; Hiraoka et al., 1998; Ramirez et al., 2000). In the case of our model, we shifted the inactivation of *I*_*rel*_ by +40*mV* and increased the slope of inactivation to fit experimental results from Li et al. (2004).

**Figure 6:**
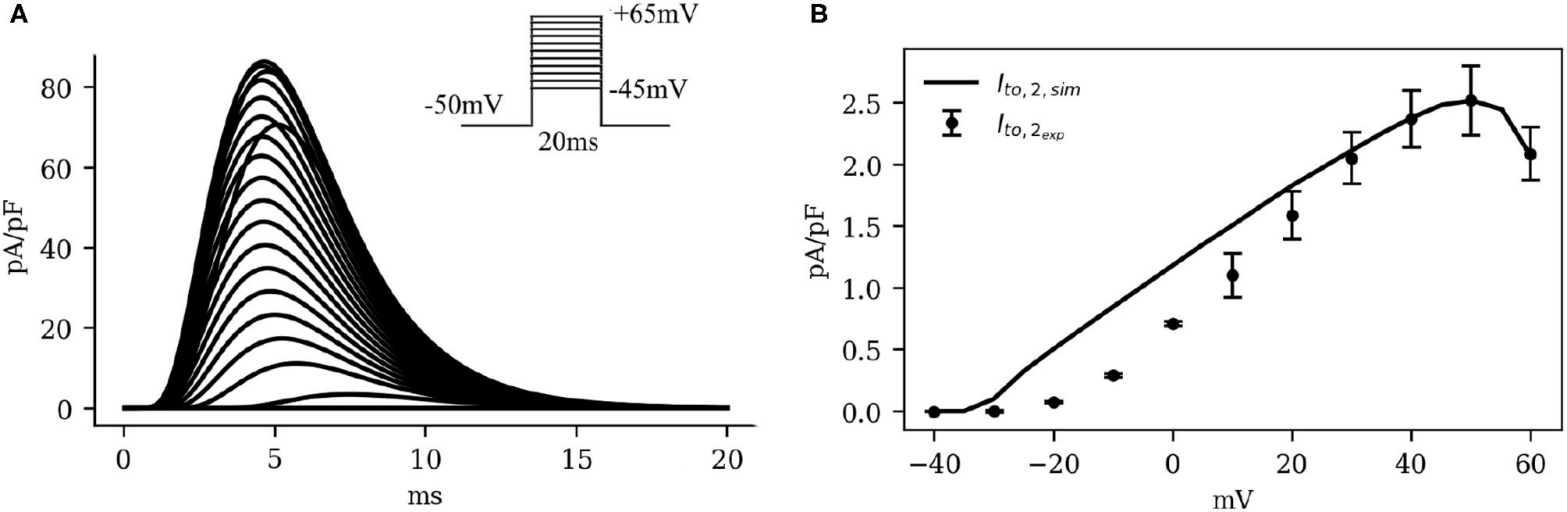
**(A)** Simulated traces (traces at 5*mV* steps from minimum to maximum in the inset) and **(B)** Current-voltage characteristics of *I*_*ClCa*_, showing experimental data (dots with error bars) from Li et al. (2004), overlaid on the model-generated curve (solid). Note the significant bell-shape of the curve at high positive voltage values.

Figure 6 shows the IV curve for *I*_*ClCa*_, as obtained using our model of the pig atrial tissue, overlaid on experimental data from Li et al. (2004). The simulated current activated earlier than in experiments, but with a good overall qualitative behavior.

#### Summary of Currents

Table 1 presents a summary of all currents adjusted in the model, along with references to the experimental data used and the parameters adjusted.

**Table 1:** Summary of currents.

**Current**	**Source**	**Species**	**Parameters adjusted**	**Experimental data source**
*I*_*Na*_	Luo and Rudy (1994)	Guinea-pig	*g*_*Na*_, τ_*m*_, τ_*h*_, τ_*j*_	our APA and *dV*/*dt*_*max*_ restitution data
*I*_*K*1_	Courtemanche et al. (1998)	Human	*g*_*K*1_, slope, half-rise, reversal potential	our RMP and *APD*_80_-*APD*_90_ restitution data
*I*_*Ca, L*_	Courtemanche et al. (1998)	Human	*g*_*Ca, L*_, *d*_∞_, τ_*d*_, τ_*f*_, τ_*j*_	*ourAPD*_20_−*APD*_50_ restitution and patch clamp data (for maximum current)
*I*_*Kur*_	Pandit et al. (2011)	Pig	*g*_*Kur, amp*_	our *APD*_10_−*APD*_30_ restitution data
*I*_*Kr*_	Courtemanche et al. (1998)	Human	*g*_*Kr*_, *x*_*r*, ∞_	Li et al. (2004) and our *APD*_60_−*APD*_90_ restitution data
*I*_*Ks*_	Courtemanche et al. (1998)	Human	*g*_*Ks*_, *x*_*s*, ∞_	Li et al. (2004) and our *APD*_60_−*APD*_90_ restitution data
*I*_*Cl, Ca*_	Ramirez et al. (2000)	Dog	*g*_*Cl, Ca*_, *w*(of *I*_*rel*_)	Li et al. (2004) and our *APD*_10_−*APD*_20_ restitution data
*I*_*NaK*_	Luo and Rudy (1994)	Guinea-pig	*I*_*NaK, max*_	our *APD*_70_−*APD*_90_ restitution data
*I*_*NaCa*_	Luo and Rudy (1994)	Guinea-pig	*I*_*NaCa, max*_	our *APD*_70_−*APD*_90_ restitution data

###  Restitution Studies

The cell model thus developed reflects ionic current properties obtained from different cell samples patched under different experimental conditions and by different groups around the world. It is unreasonable to expect that the resulting model would represent the electrophysiology of a real porcine atrial cell just by combining these currents. We need some degree of tuning to ensure that the resulting cell responds electrophysiologically in the same way as an average cell isolated from a porcine tissue sample. Therefore, we move to the next step in model development, i.e., tuning with restitution. Refining a model to ensure that it is able to reproduce the electrical properties of the heart at tissue and organ level requires detailed studies of model restitution. In cardiac electrophysiology, restitution refers to the property by which parameters such as the duration of an electrical action potential (APD) or the conduction velocity (CV) of a propagating signal vary with time between successive stimuli applied to excitable cardiac tissue. In order to study restitution, the model was extended to higher spatial dimension.

#### Spatial Extension of the Model to Higher Dimensions

To simulate wave propagation in 1 dimension and above, we added a diffusion term to Equation 1, such that the spatiotemporal evolution of the voltage is given by


(17) $$\begin{array}{r} \frac{\partial V}{\partial t}=-\frac{I_{ion}+I_{stim}}{C_{m}}+D\nabla^{2}V \end{array}$$

We used a value 0.00126 cm^2^/ms for the diffusion coefficient *D*. This choice of *D* allowed our model to reproduce the experimentally observed conduction velocity of 58 cm/s from Jang et al. (2019).

For numerical integration of Equation (17), we used a Forward Time Centered Space (FTCS) scheme, with a space differential Δ*x* = Δ*y*= 0.022 cm. The timestep chosen for the simulations was Δ*t*= 0.02 ms and all coding was done using Python or C, with MPI-based parallelization.

#### Action Potential Duration (APD)

The amount of time, during an action potential, when the membrane voltage of an excited cardiac cell is more positive than a chosen threshold, is called the action potential duration (APD) at that threshold. Typically, this threshold value is measured on the basis of degree of repolarisation of the cell membrane. Thus, APD_*X*_ refers to the amount of time during an AP, when the cell membrane is more than X% repolarised, or, less than X% depolarised.

When cardiac tissue is electrically stimulated using a train of pulses at a particular frequency, the morphology of the AP adapts to the applied pacing frequency. This reflects in the APA, RMP, ${\frac{dV}{dt}}_{max}$ and APD values at all possible levels of repolarization. Such studies are conducted to investigate the restitution behavior of the model or the tissue sample. We performed sharp-electrode measurements on pig atrial tissue to obtain APD restitution (APDR) data. We used these data to make final adjustments to the model, to perfect its electrical response to high frequency stimulation. In both experiments and simulations, pig atrial tissue was stimulated at 0.25, 0.5, 1, 2, 3, 4*Hz*, and action potentials were recorded.

We adjusted model parameters to find the most optimal parameter set that simultaneously fit each of these restitution curves with minimal deviation from measurement. Specifically, we adjusted the maximum conductance values of several currents. The final selection of conductance values is listed in Table 2.

**Table 2:** Conductance values and maximal currents after fitting restitution data.

**Conductance**	**Value (nS/pF)**
*g*_*K*1_	0.08218
*g*_*Na*_	13.9900
*g*_*Kur, amp*_	0.45539
*g*_*ClCa*_	0.15731
*g*_*Kr*_	0.01730
*g*_*Ks*_	0.0594
*g*_*Ca, L*_	0.06574
Maximal current	Value (pA/pF)
*I*_*NaK, max*_	0.94935
*I*_*NaCa, max*_	2304

**Figure 7:**
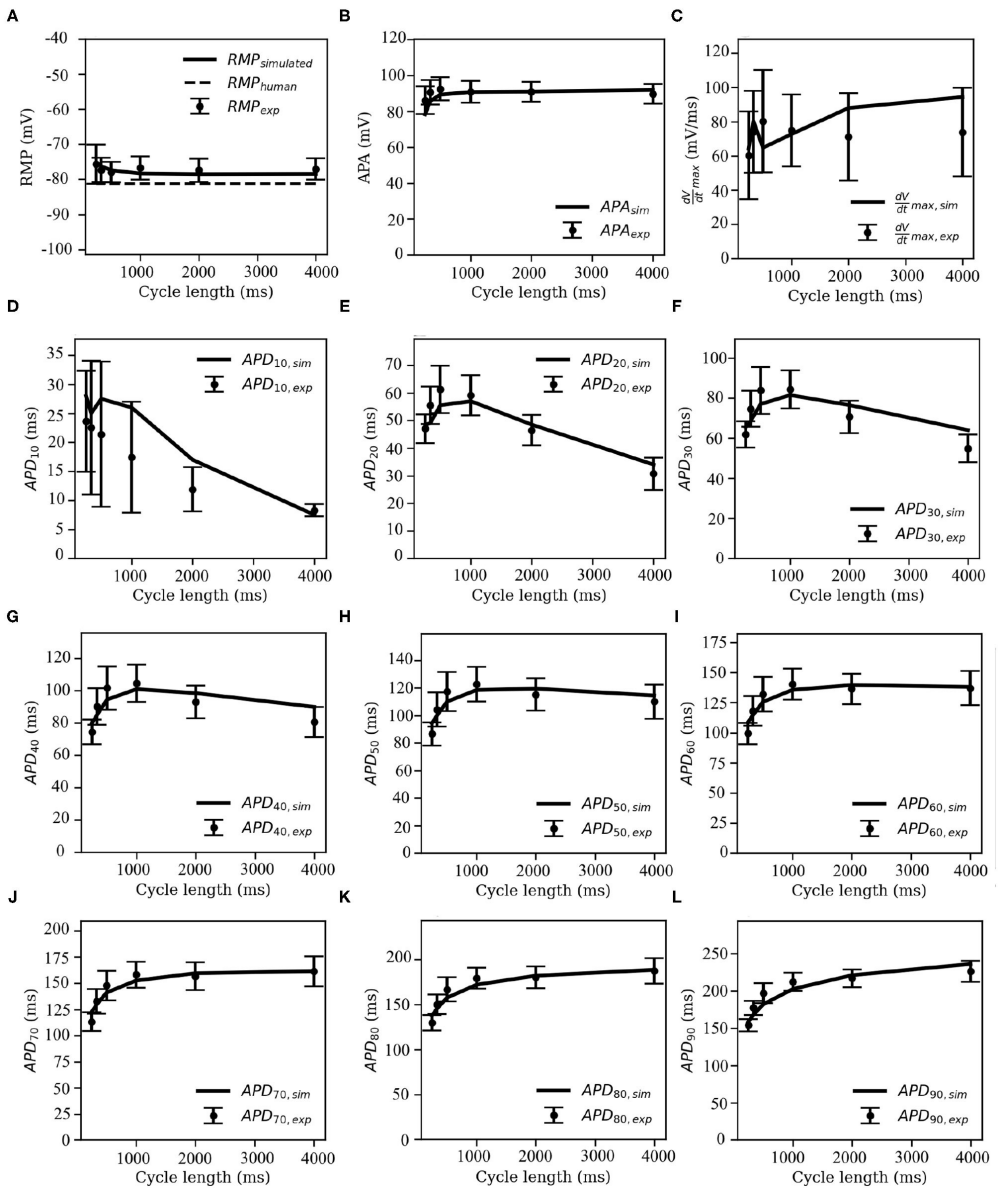
Restitution curves for **(A)** resting membrane potential (RMP), **(B)** action potential amplitude (APA), **(C)** maximum upstroke velocity (${\frac{dV}{dt}}_{max}$), and **(D–L)** action potential duration (*APD*_*X*_), at *X* = 10, 20, 30, 40, 50, 60, 70, 80, and 90% repolarisation of the membrane voltage. Solid black lines indicate the model-generated data, whereas the markers (with error bars) represent our data obtained from sharp-electrode experiments.

The overlays of our experimental and simulated data for each of the following parameters: APA, RMP, ${\frac{dV}{dt}}_{max}$, and APD_*X*_, for X = 10, 20, 30, 40, 50, 60, 70, 80, and 90% repolarization are shown in Figure 7.

Note that pig APDR curves show an interesting feature that distinguishes the model from most other mammalian species that we know of. In the early stages of repolarisation (i.e., up to APD_50_), an overall downward trend is observed for stimulation cycle lengths greater than 1,000 ms (see Figures 7D–H). This could be due to inactivation of *I*_*ClCa*_ at high pacing frequencies: slower and lesser calcium uptake causes a decrease in *I*_*ClCa*_, which significantly slows down initial AP repolarization, causing an overall higher early APD at high pacing frequencies with respect to low-frequency pacing.

**Figure 8:**
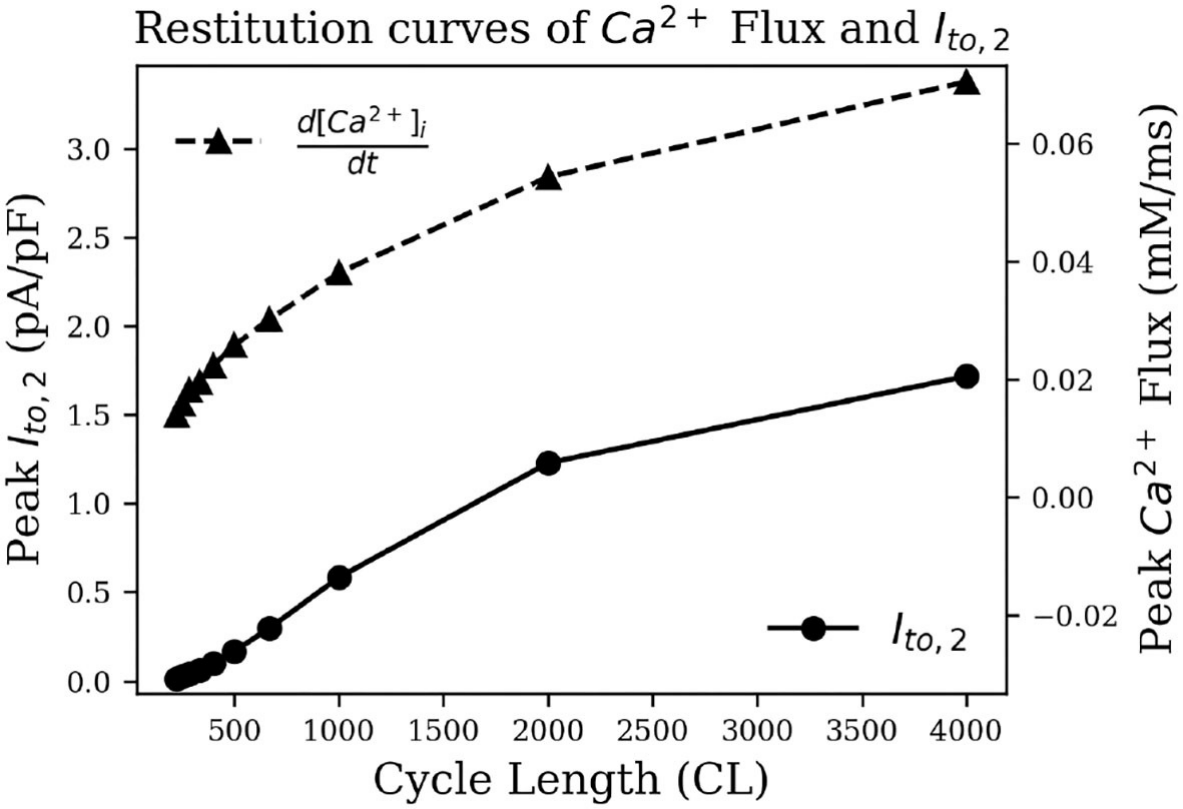
Restitution curve of peak intracellular calcium flux (dashed) and peak *I*_*ClCa*_ (solid).

To test this hypothesis, we measured intracellular calcium flux and *I*_*ClCa*_ in simulated restitution experiments. Figure 8 shows the resulting restitution curves for peak calcium flux and peak *I*_*ClCa*_ at different stimulation cycle lengths in the simulated model. A clear dependence of peak values on cycle length can be observed, and both quantities decrease significantly with a decrease in cycle length. This is indeed indicative of an initial AP repolarization phase that is heavily dependent on calcium dynamics. To the best of our knowledge, this behavior is exclusive to the pig, and might have profound implications in the translatability of studies on arrhythmia control and termination from pigs to other species.

**Figure 9:**
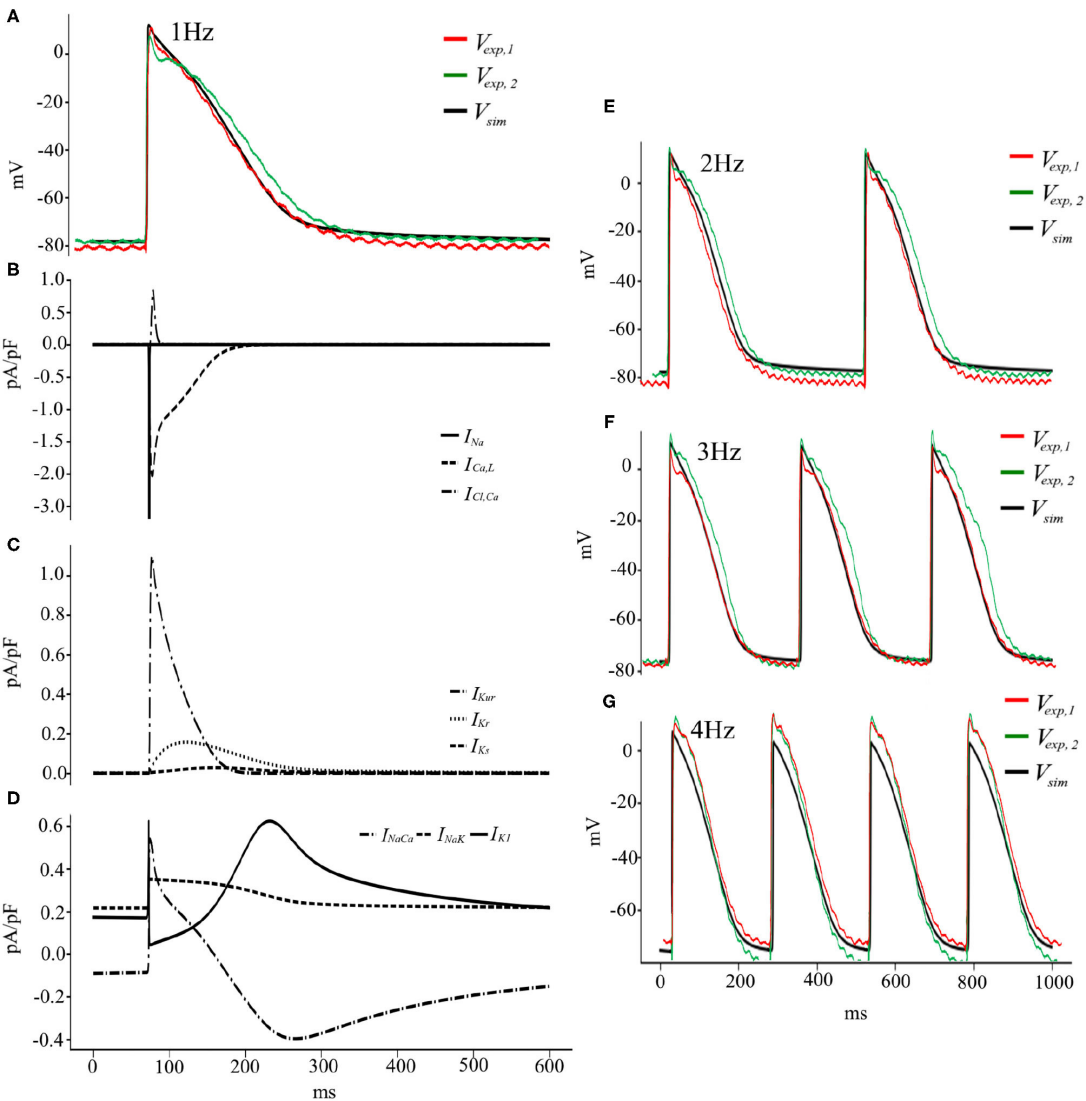
**(A)** Voltage and Current traces **(B–D)** of a pig atrial action potential at 1 Hz pacing. Simulated (solid black) and experimentally measured (green and red) traces of AP recordings from cardiac tissue. **(E–G)** Voltage traces, both simulated (black) and experimental (green and red), at pacing frequencies of 2, 3, and 4 Hz, respectively.

To summarize, the pig atrial model is capable of reproducing experimentally recorded porcine APs at different pacing frequencies within experimental deviations (Figures 9A,E–G). The discrepancies between the experimental traces show the variable nature of electrophysiology, in particular in the atria (Cherry and Fenton, 2007), and the model is good at finding a compromise and reproducing an AP with average traits within experimental tolerances. Figures 9B–D show the temporal evolution of each of the transmembrane currents considered in Equation (2). With this basic model, we now begin our study of electrical wave propagation at the tissue level.

###  Wave Propagation in 2D

**Figure 10:**
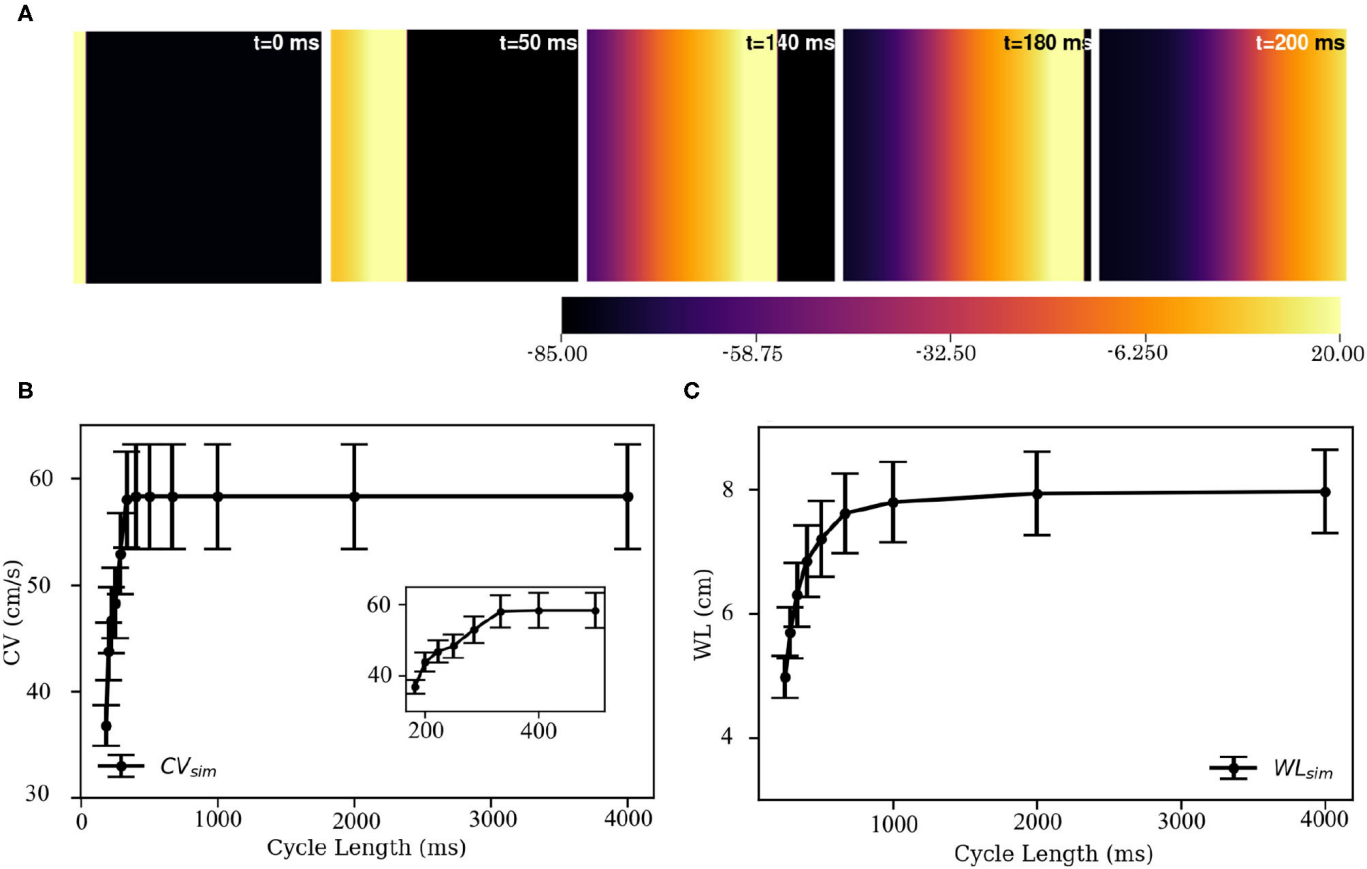
Propagation of a plane wave through simulated 2D pig atrial tissue. **(A)** Shows snapshots of the propagating wave at different times. **(B,C)** Show the conduction velocity (CV) and wavelength (WL) restitution curves, as obtained from simulations.

Electrical stimulation of a quiescent 2D domain containing pig atrial cardiomyocytes leads to propagation of an excitation wave. Our studies confirm that at frequencies below 5 Hz, the paced waves propagate with uniform and identical wavefront and waveback conduction velocity. Electrical pacing at higher frequencies does not lead to 1:1 capture. This is because the effective refractory period of the cells is approximately 215 ms. Figure 10A shows snapshots of plane wave propagation through a 2D domain containing identical pig atrial cardiomyocytes. The simulated wavelength (WL, estimated as *WL* = (*t*|_*back*, −40*mV*_−*t*|_*wavefront*_) × *CV*) and CV restitution curves are presented in Figures 10B,C.

###  Spiral Waves in the Pig Atria

#### Spiral Initiation

**Figure 11:**
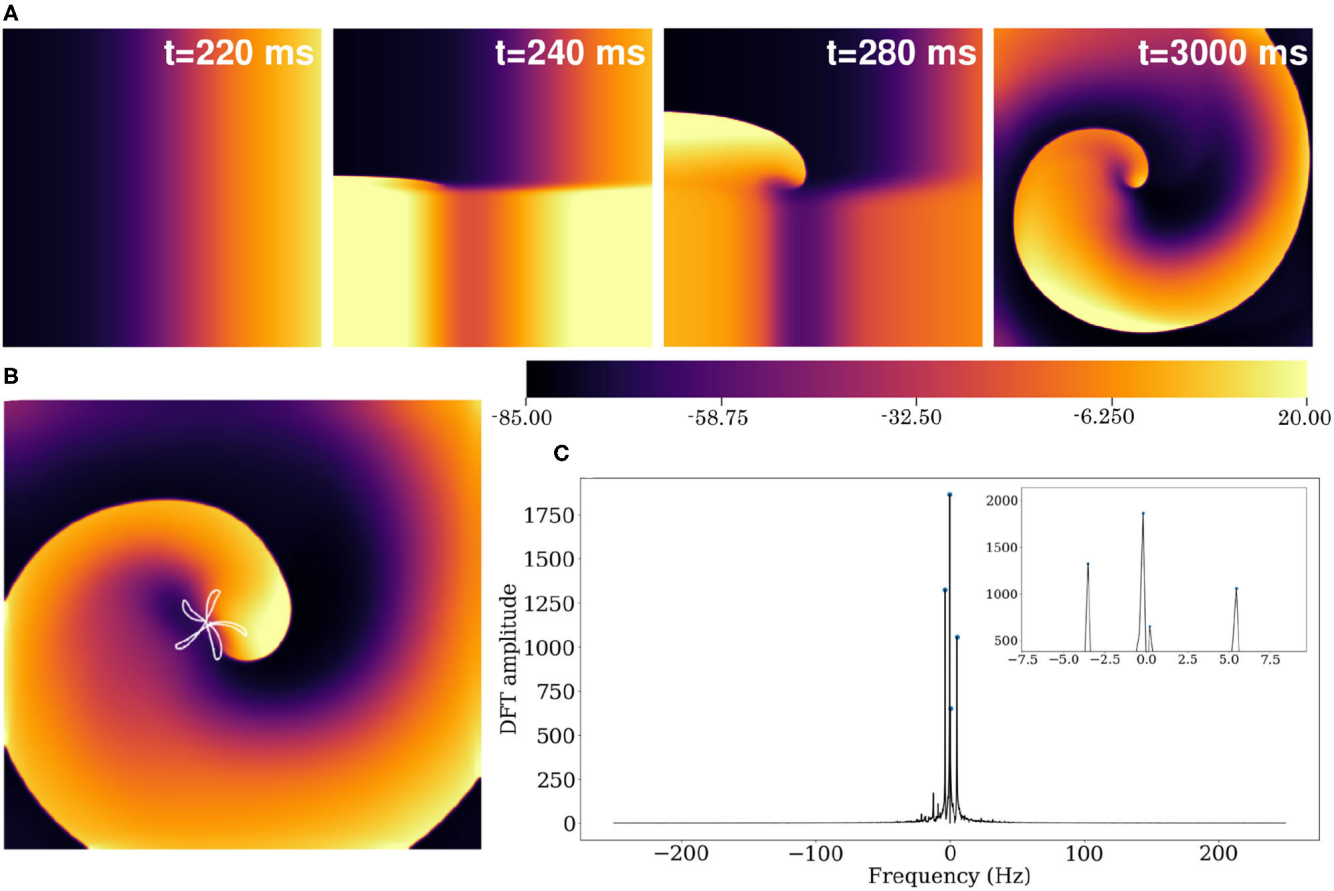
**(A)** Formation of spiral waves in a 2D pig atrial tissue of size 11.26 × 11.26 cm^2^. Here *t* = 0 is considered as the time instant at which the S1 wave is initiated (as in Figure 10A). **(B)** Hypocycloid pattern of the spiral tip trajectory. **(C)** Amplitude of the Discrete Fourier Transform (DFT) of the tip trajectory. The sampling frequency is *F*_*s*_= 500 Hz, and the corresponding resolution Δ*f*= 0.2089 Hz. The inset shows the 4 main peaks of the DFT.

Using the reported parameter set in our 2D model, we produced a spiral wave that survived for more than 40 s of simulation time. To initiate a spiral wave in a domain containing 512 × 512 grid points, we used the S1–S2 cross field protocol. We applied a line stimulus along the left edge of the domain to initiate a plane wave (S1) propagating toward the right (Figure 10A). As the waveback of the S1 wave crossed *x*= 256, a second stimulus (S2) is applied in the region *y* ≤ 256 (Figure 11A, *t* = 240 ms). This leads to propagation of the S2 wave in the region that has recovered from excitation. With time, as the wave S1 wave moves out of the domain, more excitable tissue becomes available and a spiral prototype is formed (Figure 11A, *t* = 280 ms). Figure 11A shows the spatiotemporal formation and evolution of the spiral.

#### Spiral Characterization

The spiral wave in the pig atrial tissue model meanders with a shifting hypocycloidal trajectory. The trajectory of the tip of the spiral was traced by connecting the points of intersection of the isopotential line V = −35 mV and the line dV/dt = 0 at each snapshot, spaced 10 ms apart in time. An analysis of the tip trajectory shows that the basic pattern contains 5 outward petals enclosing a center (see Figure 11B), which shifts in space at the end of every 5 rotational cycles. A Fourier analysis of the tip trajectory reveals the existence of 4 fundamental frequencies (see Figure 11C), of which *f*_0_ = 5.426*Hz* and *f*_1_ = −3.548*Hz* are the dominant ones. These contribute to the construction of the basic hypocycloidal pattern, through superposition of the two counter-rotating circular orbits at the given frequencies, where the radii of the orbits are proportional to the heights of the peaks obtained from the Fourier Transform of the trajectory.

#### Alternans

A visual impression of the spatio-temporal distribution of membrane tension during spiral wave evolution (Supplementary Video S1) indicated the occurrence of wavelength fluctuations, as opposed to a constant, uniform wavelength observed during plane wave propagation. To quantify this effect, we measured *APD*_90_ from every 8^*th*^ node within the simulation domain in X- and Y- directions. We excluded points from the region that was close to the spiral tip.

**Figure 12:**
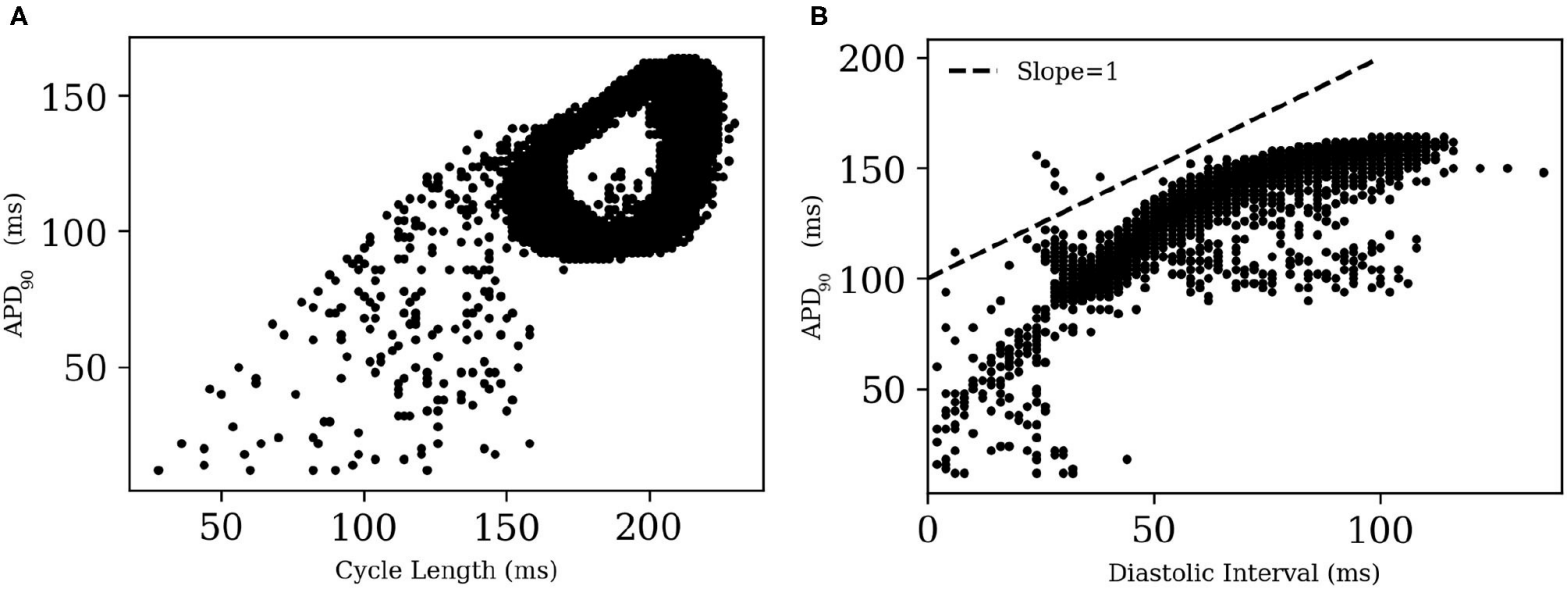
Restitution curves for *APD*_90_ in **(A)** a simulated spiral with respect to Cycle Length and **(B)** Diastolic Interval. The dashed line in **(B)** indicates the region of slope = 1.

Figure 12 shows the restitution curves of APD_90_ in the simulated spirals, both with respect to Cycle Length (CL) (Figure 12A) and Diastolic Interval (DI) (Figure 12B). Figure 12A shows the presence of alternans for cycle lengths in the range ≡ 165-215 ms. This is consistent with the restitution curve in Figure 12B, which focuses on the region with slope ≃1, a known predictor of the presence of alternans (Nolasco and Dailen, 1968). In a previous work Fakuade et al. (2021) demonstrated the occurrence of alternans at low stimulation frequencies in patients suffering from postoperative AF. Thus, our model can be used to develop useful insights into the origin and control of this alternans in pig atria.

To test if the unique current *I*_*ClCa*_ is responsible for alternans in the pig atrial model, we followed an approach that was first proposed by Gomis-Tena et al. (2003). Accordingly, we inhibited the *I*_*ClCa*_ (by 50 and 90% in two separate cases) in pig atrial model and re-initiated the spiral. However, unlike Gomis-Tena et al. (2003), alternans continued to exist in our model. APs in the simulated spiral have a duration of at most ≃ 225 ms. Referring back to Figure 8, we can see that *I*_*ClCa*_ is naturally already shut off at such small cycle lengths, and any further inactivation will obviously have a negligible effect on the behavior of the resulting spiral.

#### Spiral Wave Breakup

**Figure 13:**
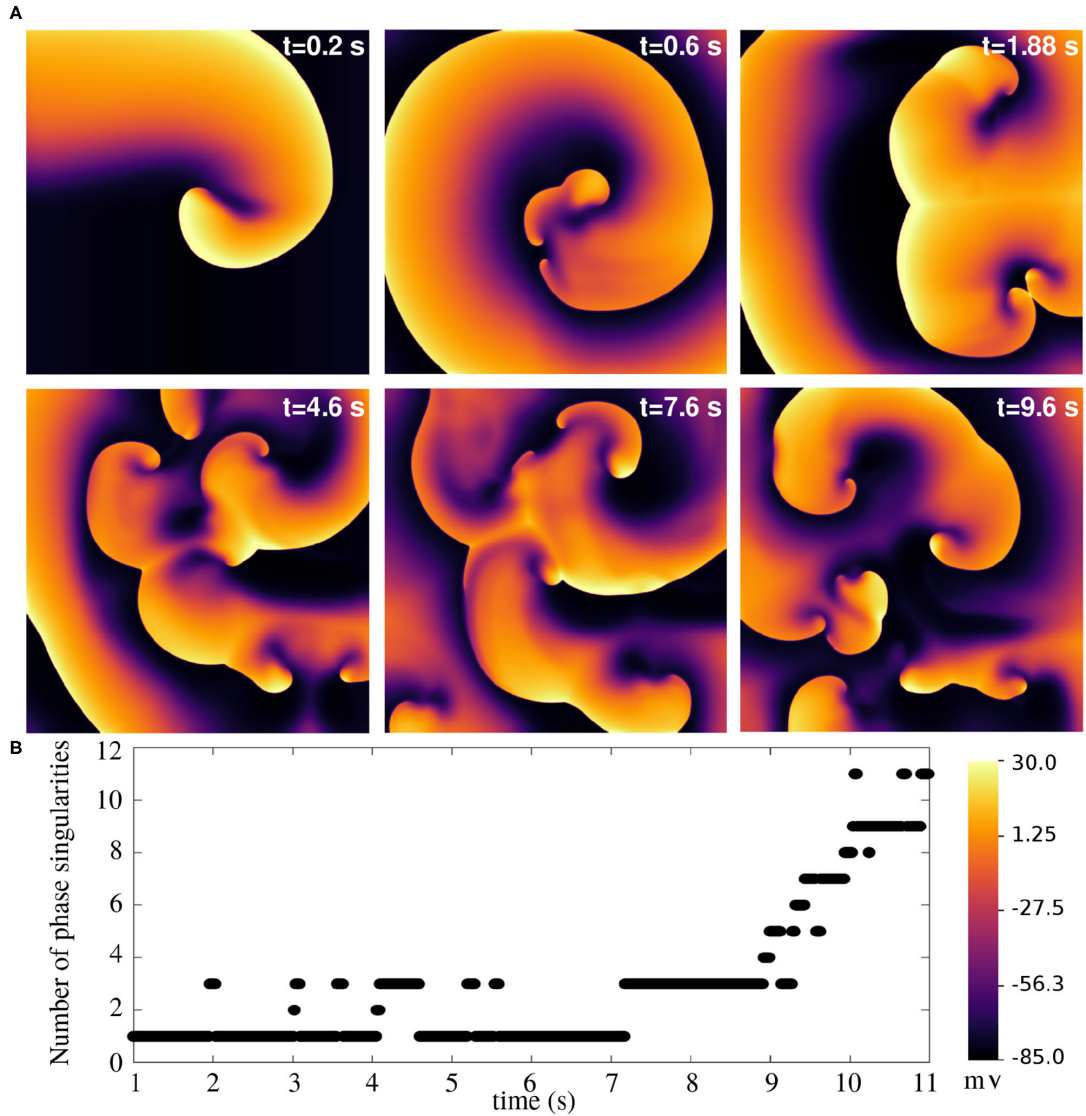
Spiral wave breakup in a 2D pig atrial tissue of size 11.26 × 11.26 cm^2^ with *G*_*Kr, max*_ reduced to 0.25x its value in the healthy pig model. **(A)** Pseudocolour plots of the membrane voltage distribution at different times demonstrates the occurrence of multiple spiral waves in the domain. **(B)** Quantification of number of spiral waves in the domain at various times, measured as the number of phase singularities (one located at each spiral tip).

Finally, we arrive at the most challenging question. Is it possible to use this model to study atrial fibrillation, with the spiral waves actually breaking up? The answer is, yes. The model does exhibit a state of sustained chaotic electrical activity in an altered parameter regime. Spiral wave breakup could be initiated by suppressing the repolarization reserve. In particular, a reduction of 75% in the value of *g*_*Kr, max*_ could lead to a state characterized by more than six spiral waves. The spatiotemporal evolution of the spiral breakup state is demonstrated in Figure 13 and in Supplementary Video S2.

## 4. Discussion

In this study, we present the first complete mathematical model of the pig atrial tissue. It is built upon experimental data on pig atria as obtained from literature, and new sharp-electrode data that was produced in our laboratory. The model is numerically stable over long timescales, and is capable of reproducing pig atrial action potentials that can be compared closely with experiments. In particular, the AP characteristics, namely APD, CV, RMP, APA, and ${\frac{dV}{dt}}_{max}$ show excellent agreement with experiments, not only for a single evoked AP, but also for the full extent of their respective restitution curves. This confirms that our model is capable of reproducing the exact electrical response as can be expected from healthy pig atrial cardiomyocytes.

Our model takes into consideration the uniqueness of the constitution of the transient outward current. In most mammalian tissue, this current is found to be predominantly *K*^+^ based. However, in pig atria, this current is solely *Cl*^−^-based and activated by the flow of *Ca*^2+^ ions. The unique dynamics of this current results in a downward trend of the early repolarization APD restitution curves; a feature that is not observed in most mammalian species. Our model reproduces this experimental trend in early APD restitution curves for large cycle lengths, and attributes the trend to the inactivation of the *I*_*ClCa*_ at low cycle lengths (Li et al., 2004).

In 2D, we demonstrate the model's ability to sustain stable spiral waves and spiral wave breakup, which adds to the suitability of the model for *in silico* studies of AF in extended media. To study spiral wave dynamics in the default model representing healthy heart tissue, we used simulation domains that are physically large compared to realistic tissue sizes. Our motivation for choosing such domains is based on the concept presented by Panfilov (2006). He showed that the pattern of stabilization of re-entries in cardiac tissue is not determined by the actual size of the heart *per se*, but by the effective size measured as heart size scaled by the wavelength of electrical activity. This means that in healthy tissue, where the wavelength of electrical activity is relatively large, it is difficult (almost impossible) to obtain self-sustaining spirals. In our default model, the wavelength of the spiral was so large that it was not possible to obtain stable spirals in tissue domains smaller than 8.5 x 8.5 cm. A Fourier analysis of the tip trajectory shows that there are 4 fundamental frequencies responsible for the dynamics of the intact spiral wave. Of these frequencies, one is associated with wave meander at *f*_*meander*_≃ 0.1 Hz, two are associated with the hypocycloid pattern, *f*_0_= 5.426 Hz and *f*_1_= 3.548 Hz, with one of those frequencies also being the frequency of rotation of the spiral arm (and thus setting the average stimulation frequency). Furthermore, our model points to the occurrence of alternans in 2D in the presence of spiral waves, between the cycle lengths of 165 ms and 215 ms. This may explain the difficulty encountered in experiments with evoking consistent APs at a pacing frequency of 5 Hz. To understand the underlying basis of this alternans, we tested an approach suggested by Gomis-Tena et al. (2003), who inhibited the *Ca*^2+^-activated *Cl*^−^ current in their canine model to inhibit alternans. Our model, however, failed to show suppression of alternans by similar inhibition of the *I*_*ClCa*_, suggesting that the alternans was not driven by the *Cl*^−^ current.

The model presented here has the same general limitations as any other ionically-detailed mathematical model of cardiac electrophysiology. We have tried to incorporate as much porcine specificity to the model as is allowed by the available experimental data. However, there are quite a few currents for which direct validation was not possible, forcing us to resort to indirect methods for model development. In our model, as listed in detail in Table 1, experimental data on current voltage characteristics was available for currents like *I*_*Kur*_, *I*_*Kr*_, *I*_*Ks*_ and *I*_*to*_. For the remaining currents, we found little or no clear information from literature. For *I*_*Na*_, we had to rely on the APA- and *dV*/*dt*_*max*_ restitution curves for obtaining the correct value of *g*_*Na*_, assuming that the channel kinetics were the same as in the human atrial cell model. For *I*_*K*1_ we relied on the RMP restitution curve and the APD restitution curves at 80–90% repolarization to formulate the current. We had no information about the *Ca*^2+^ dynamics. Therefore, it was adopted in its entirety from the Luo-Rudy dynamic model. Same applied for the pump and exchanger currents, whose maximum values we tuned based on our APD restitution data at 70–90% repolarization. Regarding the L-type *Ca*^2+^ current, the only information we had was our own data from patch-clamp recordings, which verfied that the maximum conductance used was in line with what we had chosen for the model. As previously discussed by Cherry and Fenton (2007), detailed mathematical models need to be treated with extreme considerations to appreciate their ability to correctly reproduce phenomena outside the general experimental conditions they were modeled after, and their main utility should be in developing new hypotheses in the study of already-known phenomena, rather than for the study of the dynamics of novel, unverified phenomena.

The model falls prey to the natural variability found in cardiac tissue, especially in the atria. The atrial cavities are particularly complex, more so than the ventricles, when it comes to heterogeneity and anisotropy, and the properties of cardiomyocytes are known to be affected by factors like age or sex (Cherry and Fenton, 2007). In the context of this project, this is evidently palpable in the description of *I*_*Kur*_ given in the two published papers used as sources in this model, which differ significantly, and it highlights some of the compromises that researchers must make when building a general model. In addition to intrinsic ionic heterogeneities in the heart tissue, structural factors are also known to play an important role in destabilizing reentrant electrical waves in the atria, leading to AF. A recent study by Roy et al. (2018) demonstrates how the gradients in the atrial wall thickness and tissue fibrosis can cause drifting of spiral waves across the left and right atria, resulting in AF. In another study Boyle et al. (2019) report that in patients with persistent AF who develop atrial fibrosis targeted ablation of fibrotic patches can reduce the risk of sustained AF, thereby indicating that structural heterogeneities, such as those introduced by fibrosis, play a major role in stabilizing AF.

Some of the limitations of the model come from the fact that it relies on experiments from literature for the description of individual currents, which sometimes have incomplete data (lack of information of the time dynamics of the currents, for example Li et al., 2004), or which have fundamentally different experimental setups (Li et al., 2004; Ehrlich et al., 2006). However, this model provides a good basis to start, and can be develeoped further, as and when new experimental data become available. Another important limitation of the model lies in its description of the Ca^2+^ dynamics, which is mostly taken from the human atrial model of Courtemanche et al.. The CRN model itself adapts the description of the *Ca*^2+^ dynamics from the Luo-Rudy model for guinea pig ventricular cardiomyocytes (Courtemanche et al., 1998). Thus the *Ca*^2+^-dynamics cannot be called state-of-the-art. Although it does give rise to physiologically relevant pig atrial action potentials, the model does not provide any significant insight to the fundamental role that Ca^2+^ plays in mediating I_*ClCa*_ (*I*_*to*_) and early AP repolarization. It would therefore be of great interest to make detailed experimental measurements on Ca^2+^ dynamics specific for the pig atria, with the aim of building a more accurate mathematical description to elucidate the mechanisms underlying the dynamics of *I*_*ClCa*_ and to make more accurate predictions of its behavior in arrhythmias.

Proposing the single cell model is just the first step. We have taken one step further to extend the model to 2D, where at least we can expect it to reproduce electrophysiological behavior of monolayer cell cultures. The next steps would include incorporation of natural cellular heterogeneity of cardiac tissue, together with structural heterogeneity, such as fibrosis. These are currently not addressed in our paper. Furthermore, we are trying to develop an anatomically detailed 3D atrial model of the pic heart, based on DTMRI data, which would describe the intrinsic fiber anisotropy. A study of AF in such anisotropic, realistic heart geometries would have a huge impact on the advancement of arrhythmia research.

## Data Availability Statement

The original contributions presented in the study are included in the article/Supplementary Material, further inquiries can be directed to the corresponding author/s.

## Ethics Statement

The animal study was reviewed and approved by Federation of European Laboratory Animal Science Associations (FELASA). All scientists and technicians involved have been accredited by the responsible Ethics Committee (Lower Saxony State Office for Consumer Protection and Food Safety-LAVES).

## Author Contributions

VP-Y and RM: designed and developed the model and wrote the article. TR, FF, and NV: conceived and carried out the restitution experiments. VP-Y, RM, SL, TR, FF, and NV: read and reviewed the manuscript. SL and NV: acquired funding. All authors contributed to the article and approved the submitted version.

## Funding

This work was supported by research grants from the DZHK to NV (81X4300102 and SE181) and from the Deutsche Forschungsgemeinschaft (DFG) to NV (VO 1568/3-1, VO 1568/4-1, IRTG1816, SFB1002, and under Germany's Excellence Strategy—EXC 2067/1—390729940), and to SL [German Center for Cardiovascular Research (DZHK), Project MD 28 grant no. 81Z0300403/81Z0300114 and German Research Foundation (Research Centre SFB 1002, Project C3)]. The funders had no role in study design, data collection and analysis, decision to publish, or preparation of the manuscript.

## Conflict of Interest

The authors declare that the research was conducted in the absence of any commercial or financial relationships that could be construed as a potential conflict of interest.

## Publisher's Note

All claims expressed in this article are solely those of the authors and do not necessarily represent those of their affiliated organizations, or those of the publisher, the editors and the reviewers. Any product that may be evaluated in this article, or claim that may be made by its manufacturer, is not guaranteed or endorsed by the publisher.
