# Supplementary material for: A Mathematical Model for Electrical Activity in Pig Atrial Tissue
Source: Front Physiol. 2022 Mar 10;13:812535. doi: 10.3389/fphys.2022.812535 (PMC8960738; doi:10.3389/fphys.2022.812535)
Supplement: Supplementary file 3 [file Data_Sheet_1.pdf]

Supplementary Material

1 SUPPLEMENTARY APPENDIX

Differential Equations

Temporal evolution equation for transmembrane voltage in single cell:

$$\frac{dV}{dt} = -\frac{I_{ion} + I_{stim}}{C_m} \tag{S1}$$

Total ionic current I_{ion}:

$$\begin{aligned} I_{ion} =& I_{Na} + I_{K1} + I_{ClCa} + I_{Kur} + I_{Kr} + I_{Ks} \\ &+ I_{Ca,L} + I_{p,Ca} + I_{NaK} \\ &+ I_{NaCa} + I_{b,Na} + I_{b,Ca} \end{aligned} \tag{S2}$$

Spatiotemporal evolution equation for transmembrane voltage in tissue:

$$\frac{\partial V}{\partial t} = -\frac{I_{ion} + I_{stim}}{C_m} + D\nabla^2 V \tag{S3}$$

Temporal evolution equation for all time-dependent gating variables:

$$\frac{dn}{dt} = \frac{n_\infty - n}{\tau_n} \tag{S4}$$

Temporal evolution equations for the different ionic concentrations:

$$\frac{d[Na^+]_i}{dt} = \frac{-3I_{NaK} - 3I_{NaCa} - I_{b,Na} - I_{Na}}{Fv_i} \cdot Cm \tag{S5}$$

$$\frac{d[K^+]_i}{dt} = \frac{2I_{NaK} - I_{K1} - I_{Kur} - I_{Kr} - I_{Ks} - I_{b,K}}{Fv_i} \cdot Cm \tag{S6}$$

$$\frac{d[Cl^-]_i}{dt} = \frac{I_{ClCa}}{F \cdot v_i} \cdot Cm \tag{S7}$$

$$\frac{d[Ca^{2+}]_i}{dt} = \frac{B1}{B2} \tag{S8}$$

$$B1 = \frac{2I_{NaCa} - I_{p,Ca} - I_{Ca,L} - I_{b,Ca}}{2Fv_i} \cdot Cm + \frac{v_{up}(I_{up,leak} - I_{up}) + I_{rel}v_{rel}}{v_i} \tag{S9}$$

$$B2 = 1 + \frac{[Trpn]_{max} K_{m,Trpn}}{([Ca^{2+}]_i + K_{m,Trpn})^2} + \frac{[Cmdn]_{max} K_{m,Cmdn}}{([Ca^{2+}]_i + K_{m,Cmdn})^2} \tag{S10}$$

$$\frac{d[Ca^{2+}]_{up}}{dt} = I_{up} - Iup, leak - I_{tr} \frac{v_{rel}}{v_{up}} \tag{S11}$$

$$\frac{d[Ca^{2+}]_{rel}}{dt} = (I_{tr} - I_{rel}) \left\{ 1 + \frac{[Csqn]_{max} K_{m,Csqn}}{([Ca^{2+}]_{rel} + K_{m,Csqn})^2} \right\}^{-1} \tag{S12}$$

Ionic Reversal Potential

$$E_X = (V_i - V_o) = \frac{RT}{zF} \ln(\frac{[X]_o}{[X]_i}), \quad X = Na^+, K^+, Ca^{2+}, Cl^- \tag{S13}$$

Ionic Currents

Fast Na^+ Current

$$I_{Na} = g_{Na} m^3 hj(V - E_{Na}) \tag{S14}$$

$$\begin{aligned} &\alpha_m = 0.32 \frac{V + 47.13}{1 - \exp\left[-0.1(V + 47.13)\right]}, \quad if \quad V \neq 47.13 \\ &\alpha_m = 3.2, \quad if \quad V = -47.13 \end{aligned} \tag{S15}$$

$$\tau_m = \frac{1}{\alpha_m + \beta_m} \cdot 1.7 \tag{S16}$$

$$\beta_m = 0.08 \exp\Big(-\frac{V}{11}\Big) \tag{S17}$$

$$\begin{aligned} &\alpha_h = 0.135 \exp\Big(-\frac{V + 80}{6.8}\Big), \quad if \quad V < -40 \\ &\alpha_h = 0, \quad if \quad V \geq -40 \end{aligned} \tag{S18}$$

$$\begin{aligned} &\beta_h = 3.56 \exp(0.079V) + 3.1 \cdot 10^5 \exp(0.35V), \quad if \quad V < -40 \\ &\beta_h = \left(0.13 \left[1 + \exp\Big(-\frac{V + 10.66}{11.1}\Big)\right]\right)^{-1}, \quad if \quad V \geq -40 \end{aligned} \tag{S19}$$

$$\tau_h = \frac{1}{\alpha_h + \beta_h} \cdot 2 \tag{S20}$$

$$\alpha_j = [-127140\exp(0.2444V) - 3.474\cdot 10^{-5}\exp(-0.04391V)] \cdot \frac{V+37.78}{1+\exp(0.311(V+79.23))}, \quad if \quad V < -40$$
$$\alpha_j = 0, \quad if \quad V \geq -40 \tag{S21}$$

$$\beta_j = 0.1212\frac{\exp(-0.01052V)}{1+\exp(-0.1378(V+40.14))}, \quad if \quad V < -40$$
$$\beta_j = 0.3\frac{\exp(-2.535\cdot 10^{-7})}{1+\exp(-0.1(V+32))}, \quad if \quad V \geq -40 \tag{S22}$$

$$\tau_j = \frac{1}{\alpha_j + \beta_j}\cdot 2 \tag{S23}$$

$$m_\infty = \frac{1}{1+\exp(-\frac{(V+43)}{7.7}} \tag{S24}$$

$$h_\infty = \frac{1}{1+\exp(\frac{(V+66.5)}{4.4}} \tag{S25}$$

$$j_\infty = \frac{1}{1+\exp(\frac{(V+66.5)}{4.1}} \tag{S26}$$

Inward Rectifier, $\boldsymbol{I_{K1}}$

$$I_{K1} = g_{K1}\cdot\frac{V - E_K - 5}{1+exp(0.063\cdot(V+70))} \tag{S27}$$

Transient Outward: Calcium-Driven Chloride Current, $\boldsymbol{I_{to} = I_{to,2} \equiv I_{Cl,Ca}}$

$$I_{ClCa} = g_{Cl,Ca}q_{Ca}(V - E_{Cl}) \tag{S28}$$

$$q_{Ca,\infty} = 1 - \left[\frac{1}{1+\left(\frac{F_n}{1.1e-10}\right)^3}\right] \tag{S29}$$

$$\tau_{Ca} = 2 \tag{S30}$$

Ultrarrapid Delayed Rectifier Current, $\boldsymbol{I_{Kur}}$

$$I_{Kur} = g_{Kur}\cdot u_a^3\cdot(0.25u_{i,f} + 0.75u_{i,s})\cdot(V - E_K) \tag{S31}$$

$$g_{Kur} = g_{Kur,amp}\Big[0.005 + \frac{0.05}{1+\exp(-\frac{V-15}{13})}\Big] \tag{S32}$$

$$\alpha_{u(a)} = 0.65\Bigg[\exp(-\frac{V+10}{8.5}) + \exp(-\frac{V-30}{59.0})\Bigg]^{-1} \tag{S33}$$

$$\beta_{u(a)} = 0.65\Bigg[2.5 + \exp(\frac{V+82}{17.0})\Bigg]^{-1} \tag{S34}$$

$$\tau_{u(a)} = \frac{1}{K_{Q_{10}}(\alpha_{u(a)} + \beta_{u(a)})} \tag{S35}$$

$$u_{a(\infty)} = \frac{1}{1+\exp(-\frac{V+30.3}{9.6})} \tag{S36}$$

$$\tau_{u_{if}} = 400 + 1068e^{-(\frac{V}{50})^2} \tag{S37}$$

$$\tau_{u_{is}} = 2000 + 60000e^{-(\frac{V+39.3}{30})^2} \tag{S38}$$

$$u_{i,f(\infty)} = u_{i,f(\infty)} = \frac{1}{1+\exp(\frac{(V+17.358)}{5.849})} \tag{S39}$$

Rapid Delayed Rectifier Current, $\boldsymbol{I_{Kr}}$

$$I_{Kr} = g_{Kr}\cdot x_r \cdot \frac{V-E_K}{1+exp(\frac{V-79.4825}{8.2217})} \tag{S40}$$

$$\alpha_{x,r} = 0.0003\frac{V+14.1}{1-\exp(-\frac{V+14.1}{5})} \tag{S41}$$

$$\beta_{x,r} = 7.3898\cdot 10^{-5}\frac{V-3.3328}{\exp(\frac{V-3.3328}{5.1237})-1} \tag{S42}$$

$$\tau_{x,s} = \frac{1}{\alpha_{x(r)} + \beta_{x(r)}} \tag{S43}$$

$$x_{r,\infty} = \frac{1}{1+\exp(-\frac{(V-4.445095)}{9.33047})} \tag{S44}$$

Slow Delayed Rectifier, $\boldsymbol{I_{Ks}}$

$$I_{Ks} = g_{Ks}\cdot x_s^2 \cdot (V-E_K) \tag{S45}$$

$$\alpha_{x,s} = 4 \cdot 10^{-5} \frac{V - 18.80816}{1 - \exp(-\frac{V-18.80816}{17})} \tag{S46}$$

$$\beta_{x,s} = 3.5 \cdot 10^{-5} \frac{V - 18.80816}{\exp(\frac{V-18.80816}{9}) - 1} \tag{S47}$$

$$\tau_{x,s} = \frac{1}{\alpha_{x(s)} + \beta_{x(s)}} \cdot 0.5 \tag{S48}$$

$$x_{s,\infty} = \Big[\frac{1}{1 + \exp(-\frac{(V-18.80816)}{12.6475})}\Big]^{1/2} \tag{S49}$$

L-Type Ca^{2+} Current, $I_{Ca,L}$

$$I_{Ca,L} = g_{Ca,L} d f f_{Ca}(V - 65.0) \tag{S50}$$

$$\tau_d = \frac{1 - \exp(-\frac{V+5}{6.24})}{0.035(V+5)\left[1 + \exp(-\frac{V+5}{6.24})\right]} \tag{S51}$$

$$d_\infty = \frac{1}{1 + \exp(-\frac{(V+5)}{8})} \tag{S52}$$

$$\tau_f = 9 \cdot [0.0197 \exp(-0.0337^2 (V+5)^2) + 0.02]^{-1} \tag{S53}$$

$$f_\infty = \frac{1}{1 + \exp(\frac{V+28}{6.9})} \tag{S54}$$

$$\tau_{f(Ca)} = 2, \quad f_{Ca,\infty} = \frac{1}{1 + \frac{[Ca^{2+}]_i}{0.00035}} \tag{S55}$$

NaK Pump Current

$$I_{NaK} = I_{NaK,max} f_{NaK} \frac{1}{1 + \{K_{m,Na(i)}/[Na^+]_i\}^{1.5}} \cdot \frac{[K^+]_o}{[K^+]_o + K_{m,K(o)}} \tag{S56}$$

$$f_{NaK} = \left[1 + 0.1245 \exp\Big(-0.1\frac{FV}{RT}\Big) + 0.0365\sigma \exp\Big(-\frac{FV}{RT}\Big)\right]^{-1} \tag{S57}$$

$$\sigma = \frac{1}{7}\left[\exp\Big(\frac{[Na^+]_o}{67.3}\Big) - 1\right] \tag{S58}$$

Na^+/Ca^{2+} Exchanger

$$I_{NaCa} = I_{NaCa,max} \cdot \frac{B1}{B2} \tag{S59}$$

$$B1 = \exp[\gamma VF/(RT)][Na^+]_i^3[Ca^{2+}]_o - \exp[(\gamma-1)VF/(RT)][Na^+]_o^3[Ca^{2+}]_i \tag{S60}$$

$$B2 = (K_{m,Na}^3 + [Na+]_o^3)(K_{m,Ca} + [Ca^{2+}]_o) \cdot \{1 + k_{sat}\exp[(\gamma-1)VF/(RT)]\} \tag{S61}$$

Background Currents

$$I_{b,X} = g_{b,X}(V - E_X), \quad X = Na^+, Ca^{2+} \tag{S62}$$

Ca^{2+} Pump Current

$$I_{p,Ca} = I_{p,Ca(max)}\frac{[Ca^{2+}]_i}{0.0005 + [Ca^{2+}]_i} \tag{S63}$$

Ca^{2+} Release Current from the JSR, I_{rel}

$$I_{rel} = k_{rel} \cdot u^2vw \cdot ([Ca^{2+}]_{rel} - [Ca^{2+}]_i) \tag{S64}$$

$$\tau_u = 8, \quad u_\infty = \frac{1}{1 + \exp\left(\frac{F_n - 1.367\cdot 10^{-13}}{13.67\cdot 10^{-16}}\right)} \tag{S65}$$

$$\tau_v = 1.91 + 2.09\left(1 + \exp\left[-\frac{F_n - 1.367\cdot 10^{-13}}{13.67\cdot 10^{-16}}\right]\right)^{-1} \tag{S66}$$

$$v_\infty = 1 - \frac{1}{1 + \exp\left(-\frac{F_n - 6.835\cdot 10^{-14}}{13.67\cdot 10^{-16}}\right)} \tag{S67}$$

$$\tau_w = 6.0 \cdot \frac{1 - \exp(-\frac{V-7.9}{5})}{\left[1 + 0.3\exp(-\frac{V-7.9}{5})\right](V - 7.9)} \tag{S68}$$

$$w_\infty = \frac{1}{1 + \exp(\frac{V-70}{8})} \tag{S69}$$

$$F_n = 10^{-12}v_{rel}I_{rel} - \frac{5\cdot 10^{-13}}{F}(\frac{1}{2}I_{Ca,L} - \frac{1}{5}I_{NaCa}) \cdot Cm \tag{S70}$$

Transfer Current from NSR to JSR

$$I_{tr} = \frac{[Ca^{2+}]_{up} - [Ca^{2+}]_{rel}}{\tau_{tr}} \tag{S71}$$

$$\tau_{tr} = 180 \tag{S72}$$

Ca^{2+} Uptake by the NSR

$$I_{up} = \frac{I_{up(max)}}{1 + (K_{up}/[Ca^{2+}]_i)} \tag{S73}$$

Ca^{2+} Leak Current by the NSR

$$I_{up,leak} = \frac{[Ca^{2+}]_{up}}{[Ca^{2+}]_{up(max)}} I_{up(max)} \tag{S74}$$

Calcium Buffers

$$[Ca^{2+}]_{Cmdn} = [Cmdn]_{max} \frac{[Ca^{2+}]_i}{[Ca^{2+}]_i + K_{m,Cmdn}} \tag{S75}$$

$$[Ca^{2+}]_{Trpn} = [Trpn]_{max} \frac{[Ca^{2+}]_i}{[Ca^{2+}]_i + K_{m,Trpn}} \tag{S76}$$

$$[Ca^{2+}]_{Csqn} = [Csqn]_{max} \frac{[Ca^{2+}]_{rel}}{[Ca^{2+}]_{rel} + K_{m,Csqn}} \tag{S77}$$

Numerical Integration

For the gating variables, at time t:

$$n^{(t+1)} = n_\infty - [n_\infty - n^{(t)}]e^{-\frac{\Delta t}{\tau}} \tag{S78}$$

For the rest of differential equations:

$$f^{(t+1)} = f^{(t)} + \Delta t \frac{\partial f}{\partial t} \tag{S79}$$

2 SUPPLEMENTARY TABLES

Table S1. Set of model parameters and universal constants

Parameter	Defintion	Value
R	Gas constant	8.3143 $JK^{-1}mol^{-1}$
T	Temperature	$310K$
F	Faraday constant	$96.4867C/mmol$
C_m	Membrane capacitance	$100pF$
D	Diffusion Coefficient	$0.00126cm^2/ms$
v_{cell}	Cell volume	$20100\mu m^3$
v_i	Intracellular volume	$13668\mu m^3$
v_{up}	SR uptake compartment volume	$1109.52\mu m^3$
v_{rel}	SR release compartment volume	$96.48\mu m^3$
$[K^+]_o$	Extracellular K^+ concentration	$5.4mM$
$[Na^+]_o$	Extracellular Na^+ concentration	$140mM$
$[Ca^{2+}]_o$	Extracellular Ca^{2+} concentration	$1.8mM$
$[Cl^-]_o$	Extracellular Cl^- concentration	$132mM$
g_{Na}	Maximal I_{Na} conductance	$13.9900nS/pF$
g_{K1}	Maximal I_{K1} conductance	$0.08218nS/pF$
$g_{to,2}/g_{Cl,Ca}$	Maximal I_{to} conductance	$0.15731nS/pF$
$g_{Kur,amp}$	Maximal I_{Kur} conductance	$0.45539nS/pF$
g_{Kr}	Maximal I_{Kr} conductance	$0.01730nS/pF$
g_{Ks}	Maximal I_{Ks} conductance	$0.0594nS/pF$
$g_{Ca,L}$	Maximal $I_{Ca,L}$ conductance	$0.06574nS/pF$
$g_{b,Ca}$	Maximal $I_{b,Ca}$ conductance	$0.00113nS/pF$
$g_{b,Na}$	Maximal $I_{b,Na}$ conductance	$0.000674nS/pF$
$I_{NaK(max)}$	Maximal I_{NaK}	$0.94935pA/pF$
$I_{NaCa(max)}$	Maximal I_{NaCa}	$2304pA/pF$
$I_{p,Ca(max)}$	Maximal $I_{p,Ca}$	$0.275pA/pF$
$I_{up(max)}$	Maximal I_{up}	$0.005mM/ms$
$K_{Q_{10}}$	Temperature scaling factor	3
γ	Voltage dependence parameter for I_{NaCa}	0.35
$K_{m,Na(i)}$	$[Na^+]_i$ half-saturation constant for I_{NaK}	$10mM$
$K_{m,K(o)}$	$[K^+]_o$ half-saturation constant for I_{NaK}	$1.5mM$
$K_{m,Na}$	$[Na^+]_o$ half-saturation constant for I_{NaCa}	$87.5mM$
$K_{m,Ca}$	$[Ca^{2+}]_o$ half-saturation constant for I_{NaCa}	$1.38mM$
k_{sat}	Saturation factor for I_{NaCa}	0.1
k_{rel}	Maximal release rate for I_{rel}	$30ms^{-1}$
K_{up}	$[Ca^{2+}]_i$ half-saturation constant for I_{up}	$0.00092mM$
$[Ca^{2+}]_{up(max)}$	Maximal Ca^{2+} concentration in uptake compartment	$15mM$
$[Cmdn]_{max}$	Total calmodulin concentration in myoplasm	$0.05mM$
$[Trpn]_{max}$	Total troponin concentration in myoplasm	$0.07mM$
$[Csqn]_{max}$	Total calsequestrin concentration in SR release compartment	$10mM$
$K_{m,Cmdn}$	$[Ca^{2+}]_i$ half-saturation constant for calmodulin	$0.00238mM$
$K_{m,Trpn}$	$[Ca^{2+}]_i$ half-saturation constant for troponin	$0.0005mM$
$K_{m,Csqn}$	$[Ca^{2+}]_i$ half-saturation constant for I_{up}	$0.8mM$

Table S2. Set of initial values of variables used in the model.

Variable	Value
V	-76.0
h_0	9.65×10^{-1}
d_0	1.37×10^{-4}
$x_{r,0}$	3.29×10^{-5}
$[Na^+]_i$	1.117×10^{-1}
$[K^+]_i$	1.39×10^{2}
$[Ca^{2+}]_{rel}$	1.488
$o_{i,0}$	9.99×10^{-1}
$u_{if,0}$	1
$u_{is,0}$	1
$[Cmdn]_i$	2.05×10^{-3}
$[Csqn]_i$	6.51
$[Trpn]_i$	1.18×10^{-2}
v_0	1
m_0	2.91×10^{-3}
j_0	9.78×10^{-1}
f_0	9.99×10^{-1}
$x_{s,0}$	1.87×10^{-2}
$[Ca^{2+}]_i$	0.0001013
$[Ca^{2+}]_{up}$	1.488
$[Cl^-]_i$	30
$o_{a,0}$	3.04×10^{-2}
$u_{a,0}$	4.96×10^{-3}
$f_{Ca,0}$	7.75×10^{-1}
u_0	2.35×10^{-2}
w_0	9.99×10^{-1}
I_{rel}	0
$I_{b,k}$	0
$q_{Ca,0}$	0.0000001

3 SUPPLEMENTARY VIDEOS

S1 Video.

Pseudocolour plots of the membrane voltage distribution during the formation and spatiotemporal evolution of a spiral wave. The domain contains 512×512 grid points and the video plays at 10fps. The total video represents $10s$ of simulation.

S2 Video.

Pseudocolour plots of the membrane voltage distribution during the breakup and spatiotemporal evolution of a spiral wave in altered parameter regime. Specifically, the maximum conductance for the I_{Kr} is reduced by a factor of 4. The domain contains 1024×1024 grid points and the video plays at 10fps. The total video represents $10s$ of simulation.

4 SUPPLEMENTARY CODE

To access the python source code for the pig atrial cell model, please contact the corresponding author directly.
